# Brain-Derived Neurotrophic Factor and Extracellular Vesicle-Derived miRNAs in an Italian Cohort of Individuals With Obesity: A Key to Explain the Link Between Depression and Atherothrombosis

**DOI:** 10.3389/fcvm.2022.906483

**Published:** 2022-07-13

**Authors:** Patrizia Amadio, Chiara Macchi, Chiara Favero, Marta Zarà, Giulia Solazzo, Laura Dioni, Leonardo Sandrini, Luisella Vigna, Maria Francesca Greco, Massimiliano Buoli, Cesare R. Sirtori, Angela Cecilia Pesatori, Alessandro Ieraci, Massimiliano Ruscica, Silvia Stella Barbieri, Valentina Bollati

**Affiliations:** ^1^Brain-Heart Axis: Cellular and Molecular Mechanisms Unit, Centro Cardiologico Monzino Istituti di Ricovero e Cura a Carattere Scientifico (IRCCS), Milan, Italy; ^2^Department of Biomolecular and Pharmacological Sciences, University of Milan, Milan, Italy; ^3^EPIGET LAB, Department of Clinical Sciences and Community Health, University of Milan, Milan, Italy; ^4^Occupational Health Unit, Fondazione Istituti di Ricovero e Cura a Carattere Scientifico (IRCCS) Ca' Granda Ospedale Maggiore Policlinico, Milan, Italy; ^5^Department of Neurosciences and Mental Health, Fondazione Istituti di Ricovero e Cura a Carattere Scientifico (IRCCS) Ca'Granda Ospedale, Maggiore Policlinico, Milan, Italy; ^6^Department of Pathophysiology and Transplantation, University of Milan, Milan, Italy; ^7^Department of Pharmaceutical Sciences, University of Milan, Milan, Italy

**Keywords:** BDNF, obesity, depression, atherothrombosis, EV-miRNA, cardiovascular disease

## Abstract

**Background:**

Obesity and depression are intertwined diseases often associated with an increased risk of cardiovascular (CV) complications. Brain-Derived Neurotrophic Factor (BDNF), altered in the brain both of subjects with depression and obesity, provides a potential link between depression and thrombosis. Since the relationship among peripheral BDNF, depression and obesity is not well-defined, the aim of the present report has been to address this issue taking advantage of the contribution played by extracellular vesicle (EV)-derived miRNAs.

**Research Process:**

Associations among circulating BDNF, depression and EV-derived miRNAs related to atherothrombosis have been evaluated in a large Italian cohort of obese individuals (*n* = 743), characterized by the Beck Depression Inventory (BDI-II) score.

**Results:**

BDI-II was negatively associated with BDNF levels without a significant impact of the rs6265 BDNF polymorphism; this association was modified by raised levels of IFN-γ. BDNF levels were linked to an increase of 80 EV-derived miRNAs and a decrease of 59 miRNAs related to atherosclerosis and thrombosis. Network analysis identified at least 18 genes targeted by these miRNAs, 7 of which involved in depression and CV risk. The observation of a possible link among BDNF, depression, and miRNAs related to atherothrombosis and depression in obesity is novel and may lead to a wider use of BDNF as a CV risk biomarker in this specific subject group.

## Introduction

Obesity prevalence has increased exponentially over the last few decades reaching epidemic proportions. Between 2017 and 2018, 42.4% of adult Americans were found to have a body mass index (BMI) exceeding 30 (1). Obesity is a complex condition, with serious social and psychological facets. It may affect virtually all age and socioeconomic groups and it may threaten to overwhelm the health systems of both developed and developing countries. Contrary to conventional wisdom, the obesity epidemic is not restricted to industrialized societies; in developing countries, it is estimated that over 115 million people suffer from obesity-related health problems (2).

Although in men generally there is a higher rate of overweight, women have instead higher rates of obesity, linked to numerous health consequences, including diabetes and diseases of the cardiovascular (CV) system, such as hypertension, stroke, atherosclerosis, and thrombosis (3). In addition, systematic reviews and meta-analyses have confirmed the relationship between obesity and depression, both indicating that obesity raises the risk of depression, and that depression predisposes to obesity (4). This reciprocal link establishes a potential vicious circle that can lead to or aggravate the development of metabolic disorders. In particular, it is well-known that depression increases markedly the risk to develop CV complications in obese patients (5). Understanding the biochemical bases of obesity/depression, is thus crucial in order to identify new potential biomarkers of CV diseases (CVDs).

In this complex area of research, the investigations on circulating extracellular vesicles (EVs)-derived miRNAs have achieved a great interest. In particular, changes in circulating EV-derived miRNAs have been described in atherosclerosis, thrombosis and obesity, suggesting that EVs might play a role in the crosstalk between metabolic homeostasis and CV complications (6, 7).

Genome-wide association studies (GWAS) have identified 24 potential pleiotropic genes, overlapping between mood disorders and cardiometabolic diseases (8). Among these genes, brain derived neurotrophic factor (BDNF) has been recently found in both obesity and depression (9). BDNF is a neurotrophin playing a pivotal role in several physiological and pathological conditions, including neuroplasticity, energy homeostasis, and cardiovascular function (10). It is involved in the regulation of neurogenesis and neuroprotection, food intake, atherosclerotic plaque variability and morphology, and it has been related to inflammation, fibrin clotting stability, platelet and monocyte activation, and adipocyte maturation (11–14). Altogether, the hypothesis that altered levels of this neurotrophin may predispose to a raised CV risk is supported by many Authors (15).

Polymorphisms in the BDNF gene, among these the rs6265, have been associated with depression (16) and acute coronary syndromes (17, 18); low circulating BDNF levels have been reported in coronary artery disease (12, 15, 19) and in depressed patients (20). In contrast, the relationship between peripheral BDNF and obesity is not well-defined (21). Indeed, owing to small sample sizes of clinical studies (<100 individuals), bias in subjects' recruitment, procedures of sampling/storage and BDNF measurement, as well as the lack of adjustment for confounders (e.g., platelet number, physical exercise) it is difficult to interpret results in literature (22).

Aim of the present study was, therefore, to investigate the association among BDNF, depression and EV-derived miRNAs related to atherothrombosis in a cohort of 743 obese individuals. In addition, the impact of the rs6265 BDNF polymorphism (23) on these associations has been explored.

## Materials and Methods

### Study Design and Patient Population

Seventy hundred and forty-three individuals of the cross-sectional Susceptibility to Particle Health Effects, miRNAs and Exosomes (SPHERE) study were randomly selected (24). These individuals were recruited at the Center for Obesity and Work-Activity (Fondazione IRCCS Ca' Granda Ospedale Maggiore Policlinico in Milan, Lombardy, Italy). The eligibility criteria of the SPHERE study were: (a) older than 18 years at enrolment; (b) overweight/obese according to body mass index (BMI): overweight, BMI between 25 and 30 kg/m^2^; obese: BMI of 30 kg/m^2^ or more; (c) resident in the Lombardy Region at the time of recruitment. Exclusion criteria were: diagnosis of cancer, heart diseases, stroke, other chronic diseases in the last 5 years. The study conformed to the Declaration of Helsinki and each participant provided written informed consent approved by the Ethics Committee of Fondazione IRCCS Cà Granda Ospedale Maggiore Policlinico (approval number 1425).

### Samples Collection

All patients underwent fasting blood sampling around 9 a.m. Peripheral venous blood samples were collected into vacutainer tubes containing EDTA (ethylenediaminetetraacetic acid) disodium salt (9.3 mM; Vacutainer System, Becton Dickinson, Franklin Lakes, NJ, US.); they were centrifuged at 1,200 g for 15 min. Plasma and buffy coat fractions was transferred to separate cryovials, aliquoted and immediately frozen at −80°C until use. Only one aliquot of 1.5 ml of fresh plasma was centrifuged to prepare EV pellets at 1,000, 2,000, and 3,000 g for 15 min at 4°C. The pellet was discarded to remove cell debris.

### Laboratory Measurements and Beck Depression Inventory II (BDI-II) Evaluation

Demographic, clinical and biochemical data were obtained in all subjects as previously described (25). All participants were evaluated according to the Beck Depression Inventory II (BDI-II), considered as an appropriate tool to evaluate depressive symptoms in subjects with medical comorbidities such as obesity (26). The following scores correspond to the different severity of depressive symptoms: minimal changes = 0–13, mild depression = 14–19, moderate depression = 20–28, and severe depression = 29–63.

### BDNF and PCSK9 Evaluation

Plasma BDNF and PCSK9 concentrations were measured by commercial ELISA kits (Biosensis, South Australia and R&D Systems, MN, respectively) as previously described (12, 25).

### BDNF Polymorphism

DNA was extracted from each buffy coat by the Wizard Genomic DNA purification kit (Promega, Madison, WI, USA), according to the manufacturer's instructions. BDNF polymorphism rs6265 (C/T) was selected for the current study, and analyzed using TaqMan® SNP Genotyping Assay (C_11582758_10, Applied Biosystems, San Diego, CA, USA). In a volume of 7 μl, containing 15 ng gDNA sample (3 μl), 0.35 μl TaqMan® Assay (20X), 3.5 μl TaqPath™ ProAmp™ Master Mix and 0.15 μl Nuclease-Free Water, the Polymerase Chain Reaction (PCR) was carried out as follows: pre-read 60°C for 30 s, initial denaturation 95°C for 5 min, followed by 40 cycles of denaturing at 95°C for 15 s and annealing/extension at 60°C for 60 s; a final step post-read 60°C for 30 s.The reactions and analysis were conducted on Quant Studio 12KFlex Real-Time PCR System (Applied Biosystems, San Diego, CA, USA).

### Extracellular Vesicles-miRNAs Isolation and Analysis

To prepare the EV pellets for miRNA extraction, fresh plasma deprived of cell debris was transferred to a 10.4-ml polycarbonate ultracentrifuge tube (Beckman Coulter), filled with PBS. Plasma was ultracentrifuged (BeckmanCoulter Optima-MAX-XP) at 110,000 g for 75 min at 4°C and decanted. The EV pellet was stored at −80°C until use. Isolation of miRNAs from plasma extracellular vesicles (EVs) was performed with the combination of miRNeasy kit and RNeasy Cleanup Kit (Qiagen), according to the manufacturer's protocol. MiRNAs were eluted in 20 μL of Nuclease-Free Water and stored at −80°C, until use. MiRNAs reverse transcription (RT) and preamplification reactions, followed by real-time RT-PCR analysis with the QuantStudio™ 12K Flex OpenArray® Platform (Applied Biosystems), were previously described (27). Gene Expression Suite Software (Applied Biosystems) was used to process miRNA expression data from the “TaqMan™ OpenArray™ Human MicroRNA panel” (ThermoFisher) analysis. To elucidate the possible role of the EV-miRNAs, we performed a miRNA target analysis using SpidermiR by R software (v 4.0.4). Then, we compared genes obtained by the miRNA target analysis, with genes associated with thrombosis, atherosclerosis, atherothrombosis, cardiovascular disease, and depression downloaded from DisGeNET v 7.0. Finally, genes targeted by identified miRNAs and associated with atherothrombosis and depression were used to create a network on STRING v 11.5.

### Statistical Analysis

Descriptive statistics were performed on all variables. Continuous data were expressed as the mean ± standard deviation (SD) or as the median and interquartile range (Q1–Q3), as appropriate. Categorical data were presented as frequencies and percentages. We applied univariate and multivariable negative binomial regression models for over-dispersed count observations to evaluate the relationship between BDI-II score and BDNF levels. We tested the presence of over-dispersion based upon the Lagrange Multiplier (LM) test. Continuous variables were tested for normality and linearity. Multivariable analyses were adjusted for variables significantly related to the outcome in univariate analysis (*p* < 0.05). Given the existence of multicollinearity among predictor variables (e.g., HOMA-IR, QUICKI) the variance inflation factor (VIF) statistic was calculated. To determine the best-performing model, we ran several regression equations separately, that included one or more significant explanatory variables used to predict BDI-II score. For each model, we separately estimated the respective β coefficient and standard error (SE) for each variable in the model, *p*-value, VIF statistics, as well as goodness of fit of the model (R2). Finally, the best model selected to predict the association between BDI-II score and BDNF levels was adjusted for gender, BMI, education, occupation, lifestyle, BDNF rs6265, use of antidepressant drugs, PCSK9, MAP, glycated hemoglobin, HOMA-IR, use of diabetes medications, fibrinogen, and platelets. Estimated effects were described as a percent variation associated with an increase of 1 pg/mL in BDNF concentration. The percent variation was defined as [incidence rate ratio (IRR)-1]^*^100, IRR correspond to *exp*(β).

To examine the potential effect modification by IFN-γ, we added the interaction term BDNF^*^IFN-γ to the multivariable selected model. We evaluated whether the effect of BDNF on depression score differs depending on IFN-γ concentrations. Linear regression models were used to evaluate the associations between BDNF levels and demographics and clinical characteristics of participants.

Multivariable linear regression models were applied to verify the association between miRNA expression and BDNF levels. miRNA expression values were log2-transformed to achieve a normal distribution. The regression models were adjusted for age, gender, BMI, smoking habits. Due to the high number of comparisons, we applied multiple comparison correction methods based on the Benjamini-Hochberg False Discovery Rate (FDR) to calculate the FDR *P*-value. A volcano plot of Δ% vs. –log10 *P*-values was used to display results. All statistical analyses were performed with SAS software (version 9.4; SAS Institute Inc., Cary, North Carolina, USA). A two-sided *p*-value of 0.05 was considered as statistically significant.

## Results

### Study Population

As shown in Table 1, the majority of the 743 individuals (aged 50.8 ± 13.5) were females (73.6 vs. 26.4%). Mean BMI was 33.3 ± 5.4 kg/m^2^ and the waist circumference (WC) was 100.7 ± 13.2 cm. Those not exercising physically were 60.2%, compared to 37.7% physically active. Blood pressure was in the normal range, 36.6% of participants being on hypotensive medications. The mean values of total cholesterol (TC), low-density lipoprotein cholesterol (LDL-C) and non-high-density lipoprotein cholesterol (non-HDL-C) were in the upper range of normal (TC 212.6 ± 39.8 mg/dL, LDL-C 133.4 ± 35.3 mg/dL, and non-HDL-C 153.8 ± 40.1 mg/dL). Only 10.1% of subjects were on statin medications. HDL-C and triglyceride (TG) levels were also in the normal range: mean 58.8 ± 14.9 mg/dL and median 102.2 (75.0, 144) mg/dL, respectively. Mean levels of PCSK9 were 276.3 ± 102.6 ng/mL.

**Table 1:** Demographic and clinical characteristics of the study participants (*N* = 743).

**Characteristics**	**Value**
Age, years	50.8 ± 13.5
Males	196 (26.4%)
BMI, Kg/m^2^	33.3 ± 5.4
WC, cm	100.7 ± 13.2
TC, mg/dl	212.6 ± 39.8
HDL-C, mg/dL	58.8 ± 14.9
LDL-C, mg/dL	133.4 ± 35.3
non-HDL, mg/dL	153.8 ± 40.1
LDL/HDL	2.4 ± 0.9
TC/HDL	3.8 ± 1.2
TG, mg/dL	102 [75;144]
CRP, mg/L	0.3 [0.1;0.6]
Uric acid, mg/dL	5.14 ± 1.35
Fibrinogen, mg/dL	333.5 ± 59.1
Serum creatinine, mg/dL	0.8 ± 0.3
AST, U/L	19 [16;24]
ALT, U/L	22 [16;30]
GGT, U/L	18 [13;29]
Homocysteine, μmol/L	11.2 ± 4.7
TSH, U/mL	1.8 [1.2;2.4]
Glucose, mg/dL	91 [86;100]
HbA1C, mmoL/moL	38.7 [36;42]
Insulin, U/mL	12.4 [8.5,17.8]
HOMA-IR score	2.8 [1.9;4.2]
QUICKI score	0.14 ± 0.01
PCSK9, ng/mL	276.3 ± 102.6
BDNF pg/mL	9.4 ± 5.6
Blood count	
White blood cells ( ×10^3^/μL)	6.8 ± 1.7
Red blood cells ( ×10^6^/μL)	4.8 ± 0.5
Platelets ( ×10^3^/μL)	249.3 ± 59.4
Mean Corpuscolar Volume (fL)	85.0 ± 6.4
Hemoglobin (g/dL)	13.7 ± 1.3
Hematocrit (%)	40.5 ± 3.3
Smoking status	
Never smoker	358 (48.2%)
Former smoker	261 (35.1%)
Current smoker	124 (16.7%)
Occupation
Employee	447 (60.2%)
Unemployed	63 (8.5%)
Pensioner	164 (22.0%)
Housewife	61 (8.2%)
Missing	8 (1.1%)
Education	
Primary school or less	60 (8.1%)
Secondary school	204 (27.5%)
High school	289 (38.9%)
University or more	171 (23.0%)
Missing	19 (2.5%)
Lifestyle	
Sedentary	447 (60.2%)
Physical activity	280 (37.7%)
Missing	16 (2.1%)
Heart rate, bpm	67.3 ± 10.2
Blood pressure, mmHg	
Systolic	124.9 ± 15.8
Diastolic	77.7 ± 9.9
MAP	93.4 ± 10.9
Diabetes	
Yes	85 (11.5%)
No	391 (52.6%)
Pre-diabetes	263 (35.4%)
Missing	4 (0.5%)
Antihypertensive medications	
Yes	272 (36.6%)
No	462 (62.2%)
Missing	9 (1.2%)
Statin medications	
Yes	75 (10.1%)
No	668 (89.9%)
Diabetes medications	
Yes	56 (7.5%)
No	687 (92.5%)
Antidepressant medications	
Yes	95 (12.8%)
No	648 (87.2%)
Depressive symptoms	
BDI-II score, *median [q1;q3]*	9 [5;14]
BDI-II score severity category, *n (%)*	
Minimal (0–13)	537 (72.27%)
Mild mood disturbance (14–19)	104 (14.00%)
Borderline clinical depression (20–28)	84 (11.31%)
Severe depression (>28)	18 (2.42%)

*ALT, alanine aminotransferase; AST, Aspartate aminotransferase; BDI-II, Beck Depression Inventory II; BDNF, brain-derived neurotrophic factor; BMI, body mass index; CRP, C-reactive proteins; GGT, gamma-glutamyltransferase; HbA1C, glycated hemoglobin; HDL-C, high-density lipoprotein cholesterol; HOMA-IR, omeostasis model assessment of insulin resistance; LDL-C, low-density lipoprotein cholesterol; MAP, mean arterial pressure; PCSK9, proprotein convertase subtilisin/kexin type 9; TC, total cholesterol; TG, triglyceride; TSH, thyroid stimulating hormone; WC, waist circumference; QUICKI, quantitative insulin sensitivity check index; Continuous variables are expressed as mean ± standard deviation (SD) or as median [first quartile-third quartile] if not normally distributed; discrete variables are expressed as counts (%)*.

Complete blood count (CBC), white blood cell formula, and platelets were in the normal range. Mean thyroid-stimulating hormone (TSH) levels were normal: 1.8 (1.2, 2.4) μU/ml and no participants were on thyroid substitution/suppression therapies. 16.7% were current smokers and C-reactive protein (CRP) median levels were 0.3 (0.1, 0.6) mg/L. The profile of inflammatory cytokines, namely, IFN-γ (10.4 ± 5.4 pg/mL), IL-8 (9.7 ± 9.9 pg/mL), IL-10 (4.2 [3.1;5.6] pg/mL), IL-18 (276.5 [208.5;356] pg/mL), macrophage inflammatory protein-1 α (MIP-1α) (87 [58.5;120] pg/mL), macrophage inflammatory protein-1β (MIP-1β) (186.5 [147;234] pg/mL), monocyte chemoattractant protein-1 (MCP-1) (174 [142;215.5] pg/mL), and TNF-α (5.7 [3.2;8.5] pg/mL) is reported in Supplementary Table 1.

Concerning the glycaemic control, median basal fasting plasma glucose (FPG) was 91.0 (86, 100) mg/dL, median insulin was 12.4 (8.5, 17.8) UI/ml and median glycated hemoglobin (HbA1C) 38.7 (29, 30) mmol/mol; 7.5% were on antidiabetic medications. There were no significant abnormalities in the standard laboratory tests including liver enzymes (AST, ALT, GGT).

**Figure 1:**
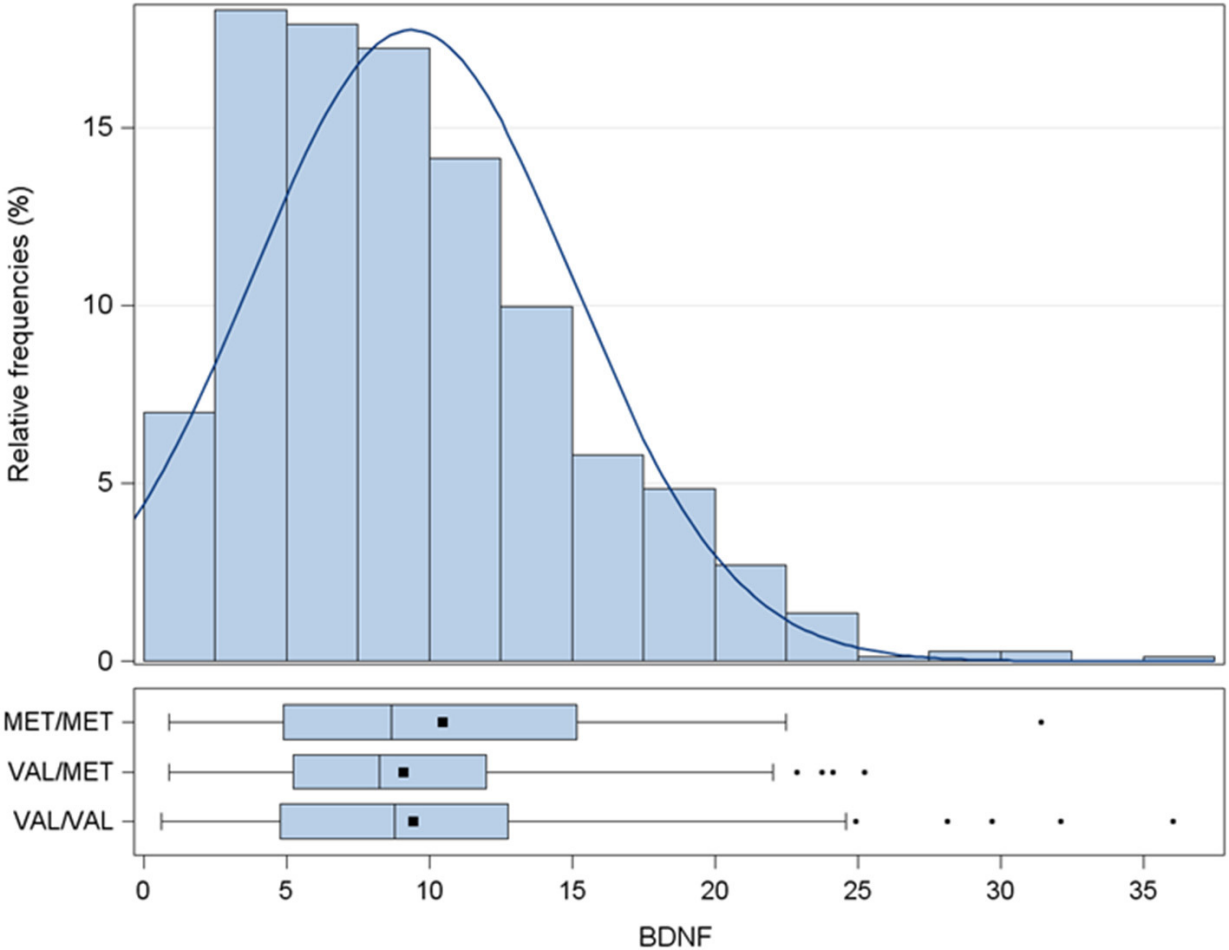
Distribution of BDNF levels (pg/mL). Data are presented as histogram and box-plot grouped by BDNF rs6265 SNP. MET/MET stands for carriers of BDNF^Met/Met^; VAL/MET stands for carriers of BDNF^Val/Met^; VAL/VAL stands for carriers of BDNF^Val/Val^. BDNF, brain derived neurotrophic factor; SNP, Single Nucleotide Polymorphism.

Average BDNF plasma concentration was 9.4 ± 5.6 pg/mL (Figure 1 and Table 1). To dissect the impact of the BDNF Val66Met variant, the entire cohort was genotyped for the rs6265 BDNF SNP. Carriers of BDNF^Val/Val^ were 62.6%, of BDNF^Val/Met^ 32.2%, and of BDNF^Met/Met^ 5.2%. The presence of the Met allele did not affect circulating levels of BDNF. These were 10.5 ± 7.2 (BDNF^Met/Met^), 9.1 ± 5.1 (BDNF^Val/Met^), and 9.4 ± 5.7 (BDNF^Val/Val^) pg/mL (*p* = 0.3503).

The median BDI-II total score was 9 (5, 14), 1.5% of participants showing severe depression. BDI-II values were higher in women compared to men, namely, 11.9 ± 7.9 and 6.8 ± 5.8, respectively (*p* < 0.0001). Less than 13% of the whole cohort was treated with antidepressant medications, namely, selective serotonin or norepinephrine/serotonin reuptake inhibitors. Antidepressant treatment was generally prescribed to subjects with moderate and severe disease, the therapeutic approach being quite homogeneously distributed among the different clusters of depression severity (Table 1).

### Relation Between BDI-II Score and Selected Study Variables

In univariate negative binomial regression analysis (Supplementary Table 2), BMI, fibrinogen, glycated hemoglobin, HOMA-IR score, QUICKI-score, PCSK9, platelets, employment status, use of antidepressant medications, were positively associated with the BDI-II score. A negative association was found for education, lifestyle, blood pressure, pre-diabetes, use of antidepressant medications and diabetes medications (Supplementary Table 2).

In the multivariate regression analysis, other variables were positively associated with the severity of depression: the HOMA-IR (β = 0.0293, SE = 0.125, *p* = 0.019), PCSK9 (β = 0.0007, SE = 0.0003, *p* = 0.0159) and platelet count (β = 0.0017, SE = 0.0005, *p* = 0.0018) were positively associated with the severity of depression (BDI-II score) (Table 2). In the univariate analysis (Supplementary Table 2), the severity of depression (BDI-II score) did not significantly associate with plasma BDNF levels (β = −0.009, SE = 0.005, *p* = 0.087), whereas this association became significant in multivariate analysis. For every one-unit increase of BDI-II, BDNF levels decreased by 1.93% (β = −0.0195, SE = 0.0056, *p* = 0.0004) (Table 3). No relationship between BDI-II score and BDNFVal66Met polymorphism was found (Supplementary Table 2).

**Table 2:** Negative binomial regression model evaluating the association between BDI-II score and BDNF adjusted for participant's characteristics.

		**β**	**SE**	**95% CI**		***P***	
Intercept		**-**	1.9327	0.4062	1.1366	2.7288	<0.0001
**BDNF**	**-**	**−0.0195**	**0.0056**	–**0.0304**	–**0.0087**	**0.0004**	
BDNF VAL66	MET/MET	0.0537	0.1343	−0.2096	0.3169	0.6896	0.6078
	VAL/MET	0.0621	0.0646	−0.0645	0.1887	0.3363	
	VAL/VAL	REF					
**PCSK9**	**-**	**0.0007**	**0.0003**	**0.0001**	**0.0012**	**0.0159**	
**Gender**	**F**	**0.4424**	**0.0791**	**0.2875**	**0.5974**	**<0.0001**	
	**M**	**REF**				
BMI	**-**	0.0056	0.0062	−0.0066	0.0178	0.3688	
MAP	**-**	−0.0053	0.0028	−0.0108	0.0001	0.0557	
Education	*Primary school or less*	0.0478	0.1391	−0.2248	0.3203	0.7312	0.1926
	*Secondary school*	0.0033	0.0805	−0.1545	0.1611	0.9674	
	*High school*	−0.1275	0.0771	−0.2786	0.0237	0.0983	
	*University or more*	REF					
Occupation	*Employee*	0.0469	0.1145	−0.1775	0.2713	0.6822	0.4386
	*Unemployed*	0.2072	0.1455	−0.0780	0.4924	0.1544	
	*Pensioner*	0.0684	0.1225	−0.1717	0.3084	0.5769	
	*Housewife*	REF					
Lifestyle	*Sedentary*	0.0929	0.1247	−0.1515	0.3372	0.4563	0.2029
	*Active*	−0.0242	0.1264	−0.2720	0.2236	0.8482	
	*Sporty*	REF					
**Diabetes medications**	**Yes**	**0.2928**	**0.1317**	**0.0347**	**0.5510**	**0.0262**
	**No**	**REF**				
**Use of antidepressant medications**	**Yes**	**0.3400**	**0.0871**	**0.5107**	**0.1694**	**<0.0001**
	**No**	**REF**				
HOMAIR	-	0.0293	0.0125	0.0048	0.0539	0.0192
Glycated hemoglobin	-	0.0014	0.0041	−0.0066	0.0095	0.7248
Fibrinogen	-	−0.0001	0.0005	−0.0012	0.0009	0.8201
Platelets	-	0.0017	0.0005	0.0006	0.0027	0.0018
Dispersion		0.3441	0.0285	0.2926	0.4048		

*BDNF, brain-derived neurotrophic factor; BMI, body mass index; HOMA-IR, omeostasis model assessment of insulin resistance; MAP, mean arterial pressure; PCSK9, proprotein convertase subtilisin/kexin type 9. Values in bold represent significant changes*.

**Table 3:** Slope coefficients from univariate linear regression models to evaluate association between BDNF and demographics and clinical characteristics of participants.

**Independent variable**	**β**	**SE**	**95% CI**		***P***	
BDNF polymorphism						0.3488
Val/Val	**REF**	**-**	**-**	**-**	**-**	
Val/Met	−0.328	0.446	−1.203	0.547	0.4624	
Met/Met	1.045	0.935	−0.788	2.877	0.2639	
Age. Years	−0.007	0.015	−0.037	0.023	0.6411	
Gender					0.1234	
Females	0.719	0.467	−0.196	1.634		
Males	**REF**	**-**	**-**	**-**		
BMI, kg/m^2^	−0.043	0.038	−0.118	0.031	0.2568	
**TC, mg/dL**	**0.016**	**0.005**	**0.006**	**0.026**	**0.0020**	
HDL-C, mg/dL	0.003	0.014	−0.025	0.030	0.8472	
**LDL-C, mg/dL**	**0.014**	**0.006**	**0.003**	**0.026**	**0.0134**	
**non-HDL-C, mg/dL**	**0.015**	**0.005**	**0.005**	**0.026**	**0.0026**	
LDL-C/HDL-C	0.397	0.230	−0.053	0.848	0.0838	
**TC/HDL-C**	**0.370**	**0.185**	**0.008**	**0.732**	**0.0449**	
**TG, mg/dL**	**0.007**	**0.003**	**0.002**	**0.012**	**0.0077**	
CRP, mg/L	0.171	0.288	−0.392	0.735	0.5514	
Uric acid, mg/dL	−0.092	0.154	−0.393	0.210	0.5512	
Fibrinogen, mg/dL	−0.002	0.004	−0.009	0.005	0.4920	
Serum creatinine. mg/dL	−0.918	0.654	−2.201	0.365	0.1608	
AST, U/L	−0.028	0.025	−0.077	0.020	0.2510	
ALT, U/L	0.002	0.012	−0.022	0.025	0.8726	
**GGT, U/L**	**0.019**	**0.009**	**0.001**	**0.038**	**0.0396**	
Homocysteine, μmol/L	0.054	0.044	−0.032	0.140	0.2145	
TSH, U/mL	0.047	0.137	−0.222	0.316	0.7304	
Glucose, mg/dL	0.024	0.024	−0.023	0.071	0.3182	
HbA1C, mmoL/moL	0.011	0.008	−0.004	0.027	0.1506	
Insulin level, U/mL	0.002	0.021	−0.039	0.042	0.9352	
HOMAIR score	0.016	0.068	−0.116	0.149	0.8080	
QUICKI score	−19.416	15.842	−50.466	11.633	0.2203	
PCSK9, ng/mL	−0.002	0.002	−0.007	0.002	0.3305	
Blood count						
**White blood cells**	**0.289**	**0.123**	**0.049**	**0.530**	**0.0184**	
**Red blood cells**	**0.956**	**0.453**	**0.068**	**1.845**	**0.0349**	
**Platelets**	**0.032**	**0.003**	**0.025**	**0.038**	**<0.0001**	
Mean Corpuscolar Volume	−0.039	0.032	−0.102	0.025	0.2293	
Hemoglobin	0.111	0.157	−0.197	0.419	0.4812	
Hematocrit	0.068	0.062	−0.054	0.191	0.2730	
Cytokines						
IFN-γ	−0.019	0.033	−0.084	0.046	0.5727	
IL-8	0.038	0.021	−0.003	0.079	0.0677	
IL-10	−0.005	0.007	−0.019	0.008	0.4487	
IL-18	−0.001	0.001	−0.003	0.002	0.6056	
MIP-1α	0.002	0.004	−0.006	0.009	0.6580	
**MIP-1β**	**0.004**	**0.002**	**0.001**	**0.008**	**0.0172**	
**MCP-1**	**0.011**	**0.002**	**0.008**	**0.015**	**<0.0001**	
TNF-α	−0.007	0.011	−0.029	0.015	0.5316	
Smoking status						0.1608
Former smoker	−0.224	0.456	−1.118	0.670	0.6233	
Current smoker	0.925	0.584	−0.219	2.070	0.1130	
Never smoker	REF	-	-	-		
Occupation						0.5542
Unemployed	0.006	0.754	−1.471	1.483	0.9938	
Pensioner	−0.044	0.511	−1.046	0.958	0.9309	
Housewife	−1.091	0.764	−2.589	0.407	0.1535	
Employee	REF	**-**	**-**	**-**		
Education						0.8123
Secondary school	0.578	0.821	−1.032	2.188	0.4816	
High school	0.526	0.794	−1.029	2.081	0.5075	
University or more	0.810	0.839	−0.835	2.455	0.3346	
Primary school or less	REF	**-**	**-**	**-**		
Lifestyle						
Active	0.047	0.456	−0.846	0.940	0.9173	
Sporty	−0.438	0.865	−2.132	1.257	0.6128	0.8627
Sedentary	REF	**-**	**-**	**-**		
Heart rate. *bpm*	0.029	0.020	−0.011	0.068	0.1552	
Blood pressure. *mmHg*						
Systolic	−0.002	0.013	−0.028	0.023	0.8496	
Diastolic	0.007	0.021	−0.034	0.048	0.7274	
MAP	0.002	0.019	−0.035	0.040	0.9046	
Diabetes						0.1569
Yes	0.498	0.670	−0.816	1.811	0.4577	
Pre-diabetes	0.854	0.447	−0.021	1.729	0.0558	
No	REF	**-**	**-**	**-**		
Antihypertensive medications					0.3249	
Yes	−0.424	0.430	−1.267	0.420		
No	REF	-	-	-		
Antidepressant medications					0.7968	
Yes	−0.159	0.617	−1.368	1.050		
No	REF	**-**	**-**	**-**		
Statin medications					0.3478	
Yes	−0.642	0.684	−1.982	0.698		
No	REF	-	-	-		
Diabetes medications						
Yes	0.777	0.780	−0.752	2.305	0.3192	
No	REF	**-**	**-**	**-**		

*ALT, alanine aminotransferase; AST, Aspartate aminotransferase; BDNF, brain-derived neurotrophic factor; BMI, body mass index; CRP, C-reactive proteins; GGT, gamma-glutamyltransferase; HbA1C, glycated hemoglobin; HDL-C, high-density lipoprotein cholesterol; HOMA-IR, omeostasis model assessment of insulin resistance; IFN-γ, interferon gamma; IL, interleukin; MCP-1, monocyte chemoattractant protein-1; MIP-1α, macrophage inflammatory protein-1 alpha; MIP-1β, macrophage inflammatory protein-1 beta; LDL-C, low-density lipoprotein cholesterol; PCSK9, proprotein convertase subtilisin/kexin type 9; TC, total cholesterol; TG, triglyceride; TNF-α, tumor necrosis factor-α. Values in bold represent significant changes*.

Similarly, the association between BDI-II score and the use of antidepressants and of diabetes medications were robust even after adjusting for the relevant covariates (Table 2).

### Role of Inflammatory Markers in the Association Between Plasma BDNF Levels and BDI-II Score

**Figure 2:**
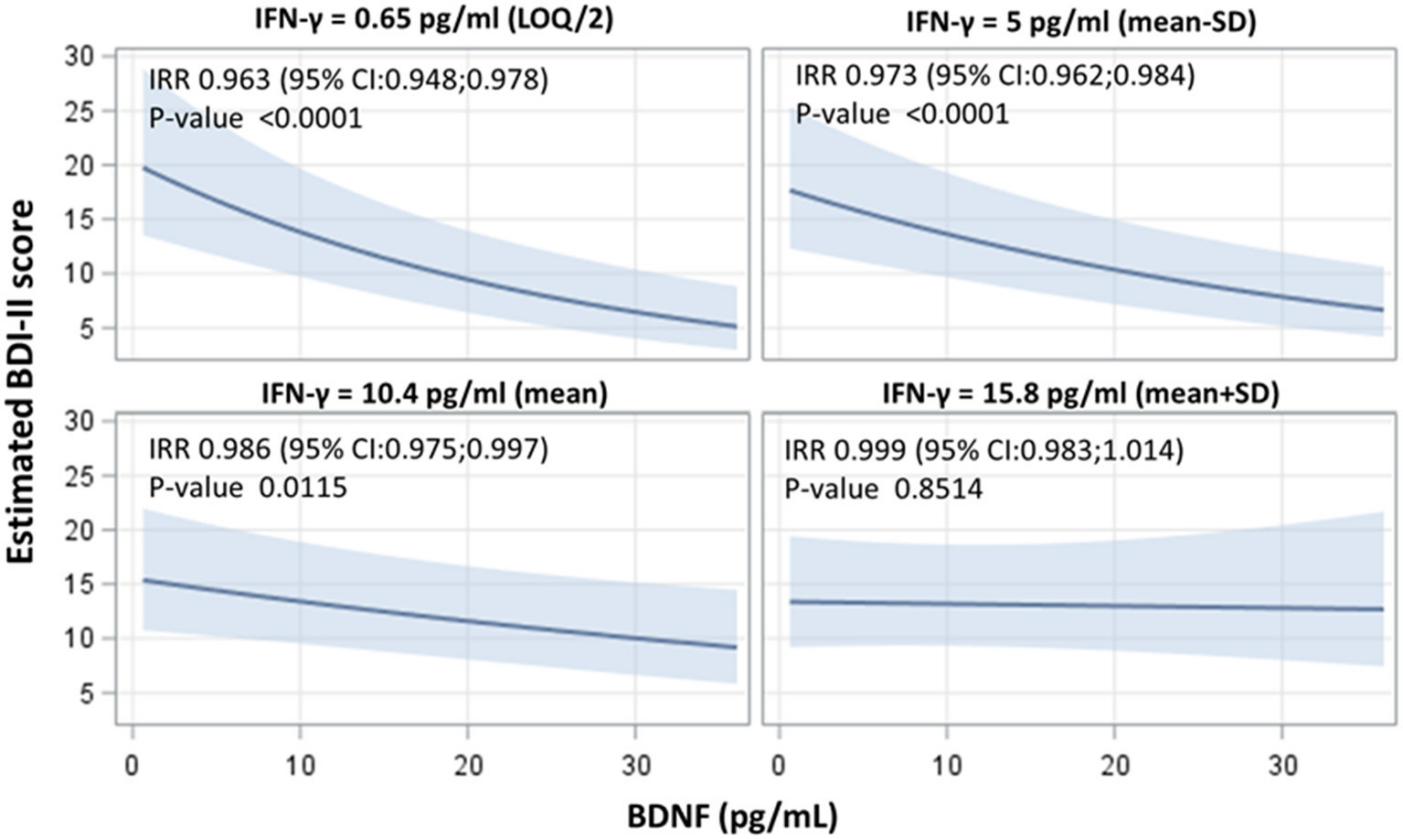
Interactive effects of BDNF and IFN-γ on the BDI-II score, indicating the strength of association between BDNF concentrations and BDI-II score at four selected levels of IFN-γ (LOQ/2), mean - standard deviation (*SD*), mean and mean ± *SD* value). Adjusted incidence rate ratios (IRR) were reported for one unit increase in BDNF concentrations, at each IFN-γ level. IRR correspond to *exp*(β); BDNF, Brain-Derived Neurotrophic Factor; BDI-II, Beck Depression Inventory-II; IFN-γ, interferon gamma.

Since inflammation has been linked to depression (28), the impact of the inflammatory environment on the previous associations was evaluated. Among the different tested cytokines (Supplementary Table 1), IFN-γ significantly modified the association between BDNF and BDI-II (interaction term *p*-value = 0.011). The strength of the association was modified in different IFN-γ strata. Each unit rise in BDNF led to an incidence rate ration (IRR) of 0.963 (95% CI 0.948–0.978), of 0.973 (95% CI 0.962–0.984), of 0.986 (95% CI 0.975–0.0997), and of 0.999 (95% CI 0.983–1.014), respectively, at IFN-γ levels of 0.65, 5, 10.4 and 15.8 pg/mL (Figure 2). No significant association between the different tested cytokines and BDI-II score was found (Supplementary Table 2).

### Association Between BDNF and CV Risk Factors

BDNF levels were positively associated with CV parameters: TC (β = 0.016, SE 0.005, *p* = 0.0020), LDL-C (β = 0.014, SE = 0.006, *p* = 0.0134), non-HDL-C (β = 0.015, SE = 0.005, *p* = 0.0026), and TG (β = 0.007, SE = 0.005, *p* = 0.0077). In addition, a positive association between BDNF and platelets (β = 0.032, SE = 0.003, *p* < 0.0001), white (β = 0.289, SE = 0.123, *p* = 0.0184), and red (β = 0.956, SE = 0.453, *p* = 0.0349) blood cells were found. Among the tested proinflammatory cytokines, MIP-1β (β= 0.004, SE = 0.002, *p* = 0.0172) and MCP-1 (β= 0.011, SE = 0.002, *p* < 0.0001) were positively associated with plasma BDNF levels (Table 3).

### EVs-Derived miRNA, Atherothrombosis, and Depression

**Figure 3:**
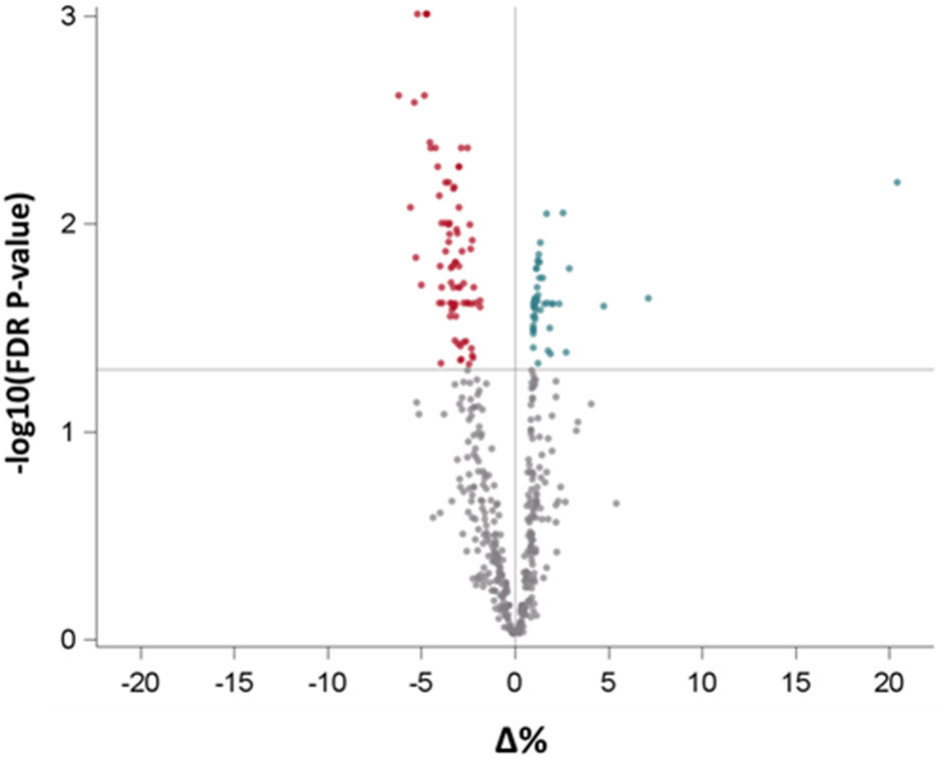
Volcano plot reporting the association of BDNF levels with all measured miRNAs in extracellular vesicles. Red and green dots represent miRNAs, respectively, with negative and positive statistically significant association with BDNF. BDNF, Brain-Derived Neurotrophic Factor.

To further dissect the possible involvement of BDNF in CV disease, we analyzed EV-derived miRNAs isolated from the same individuals. After data cleaning, 508 miRNAs were expressed in at least one subject. In a model adjusted for age, gender, BMI, and smoking habit, BDNF levels were associated with a significant increase of 80 miRNAs (the up-regulated group) and a significant decrease of 59 miRNAs (the down-regulated group), after FDR adjustment for multiple comparisons (FDR *p* < 0.05; Figure 3 and Supplementary Table 3).

By using the DisGeNET database, we identified the genes targeted by these 139 miRNAs specifically related to thrombosis, atherosclerosis, atherothrombosis and CV disease. Among the group of up-regulated miRNAs targeted genes (11,890), 1,091 were associated with CV disease, 1,253 with atherosclerosis, 60 with thrombosis, and 69 with atherothrombosis (Supplementary Figure 1). In particular, they represented more than 50% of total genes included in the databases of atherothrombosis and more than 60% of those included in the databases of thrombosis, atherosclerosis and CV disease.

Among the group of down-regulated miRNAs targeted genes (11,121), 1,008 were associated with CV disease, 1,180 with atherosclerosis, 50 with thrombosis, and 63 with atherothrombosis (Supplementary Figure 2). In particular, they represented between 50 and 57% of total genes included in the four datasets. Among all the identified target genes, 18 were reported in all four datasets (thrombosis, atherosclerosis, atherothrombosis, and cardiovascular disease). Specifically, 15 target genes were associated with the group of up-regulated miRNAs and 13 target genes with the down-regulated group. The following 10 target genes CYP2C19, F3, IL6, MTHFR, PLAT, PTGS 2, SERPINE 1, TFP1, THBD, and TNF were shared between the up- and down-regulated groups (Supplementary Table 4).

**Figure 4:**
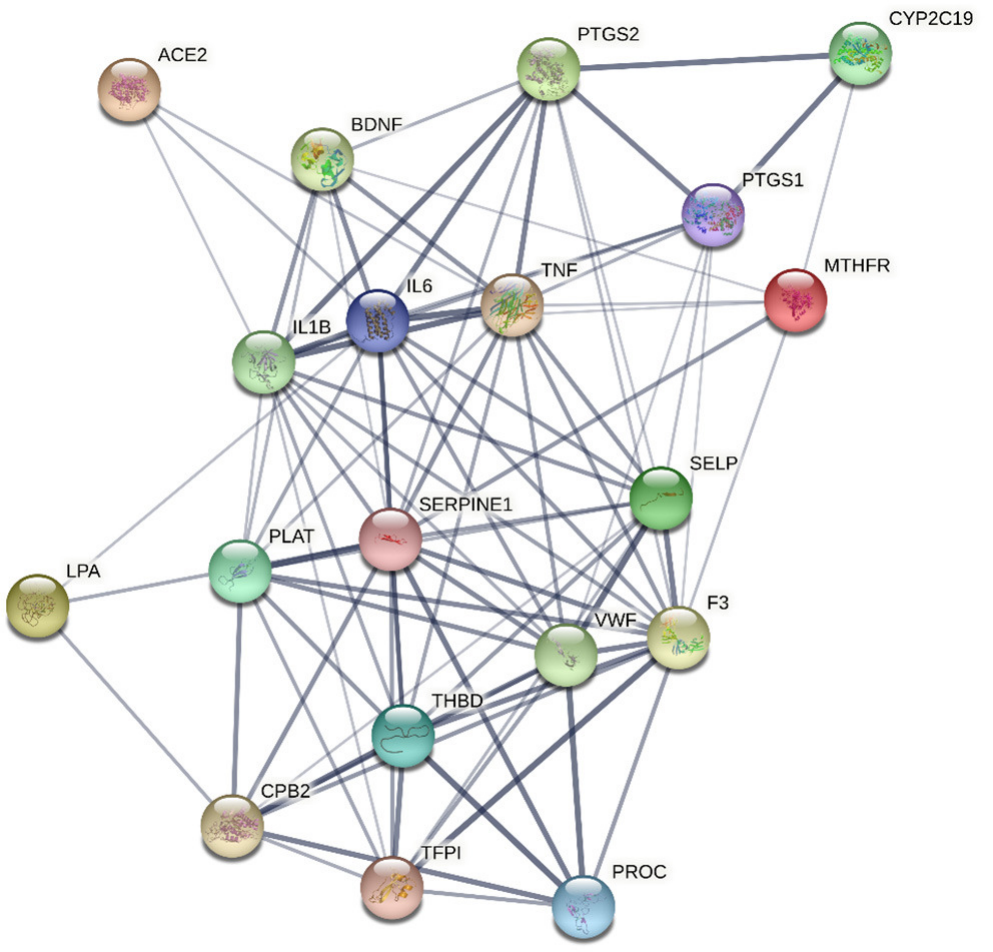
Network, constructed by the STRING protein-protein interaction network tool, of the 18 genes targeted by miRNAs associated with Brain-Derived Neurotrophic Factor and related to thrombosis, atherosclerosis, atherothrombosis, and cardiovascular disease. The line thickness represents strength of the interaction.

*In silico* multiple-protein interaction analysis (STRING) revealed that all 18 target genes (AC2, PTGS2, CYP2C19, IL18, IL6, TNF, PTGS1, MTHFR, LPA, PLAT, SERPINE1, SELP, F3, VWF, THBD, CPB2, TFPI, and PROC) directly or indirectly interacted with BDNF (Figure 4). Overall, these data support the important relationship between circulating BDNF levels and increased CV risk in this population.

**Figure 5:**
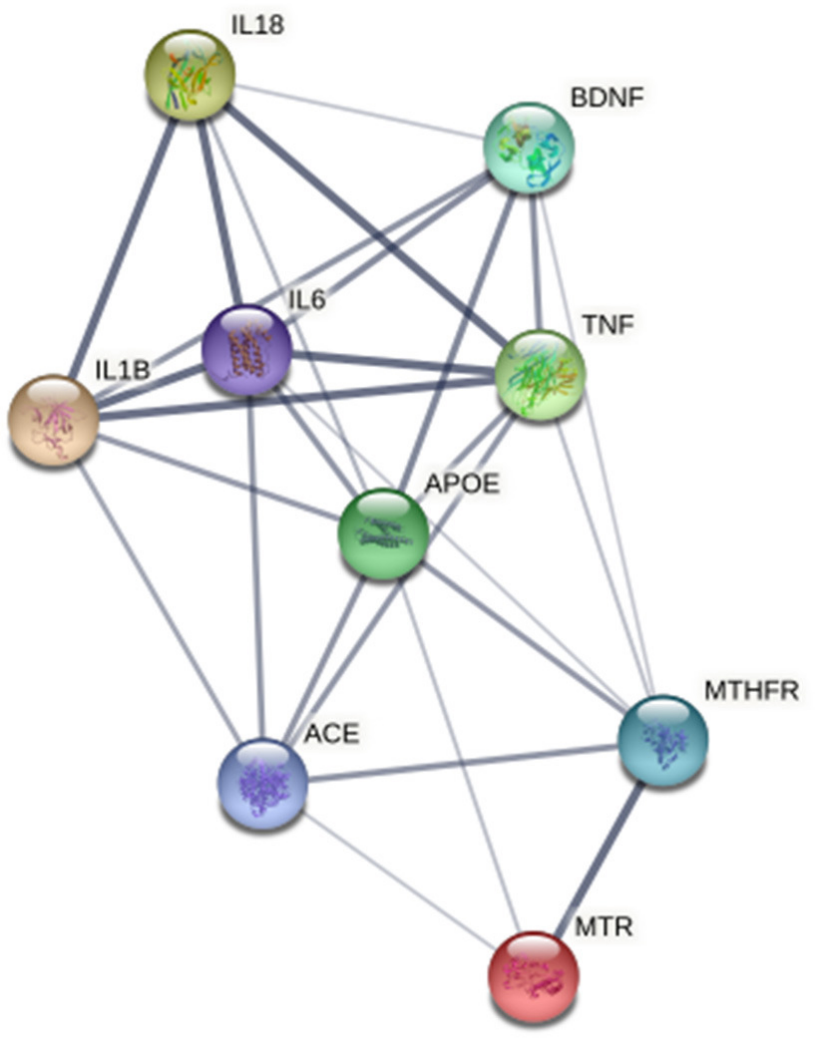
Network, constructed by the STRING Protein–Protein interaction network tool, depict interactions, among the 7 genes targeted by miRNA associated with Brain-Derived Neurotrophic Factor and related to both atherothrombosis and depression. The line thickness represents strength of the interaction.

Finally, in order to dissect the potential relationship among BDNF, obesity, atherothrombosis and depression, we performed a further analysis by using specific datasets of the DisGeNET pertaining to depression, i.e., chronic depression, clinical depression, mild depression, recurrent depression and severe depression. We found that 51 out of 11,890 genes targeted by up-regulated miRNAs were associated with depression, of which 8 were reported also in the atherothrombosis database (Supplementary Table 5). Regarding the genes targeted by down-regulated miRNAs, 52 out of 11,121 were related to depression, 7 of which were also found in the atherothrombosis dataset (Supplementary Table 6). Interestingly, BDNF emerged among the genes shared by depression and atherothrombosis, and it was well-connected to all the genes identified in both databases, namely, IL-1β, IL-6, IL-18, TNF, APOE, ACE, MTR, MTHFR genes (Figure 5).

## Discussion

In the present study by using data from an Italian cohort of obese individuals, we identified a significant negative association between depression scores and BDNF plasma levels, a finding that was modified by elevated inflammatory levels of IFN-γ. In addition, we provided evidence that the amount of circulating BDNF can reflect the EV-derived miRNAs related to CV disease and depression, supporting the hypothesis that BDNF may be the link between mood disorders and cardiometabolic diseases. Depression development is linked to several factors among which obesity most likely has a causal role. Mendelian randomization studies have provided clear evidence that high BMI is causally linked to the likelihood to develop depression (31).

Among central nervous system (CNS) determinants of obesity, BDNF could exert a potentially significant role. The relationship of BDNF with obesity and depression has been the subject of many investigations which collectively described a reduction of brain BDNF levels in both obesity and depression. Indeed, by contributing to the regulation of both synaptic plasticity and energy metabolism, including feeding behavior, BDNF has been recognized as a key target in the relationship between metabolic syndrome and psychiatric diseases. BDNF by activation of tyrosine kinase receptor-B (Trk-B) promotes neuronal survival, neuroplasticity, synaptogenesis, and survival and differentiation of serotonergic neurons through different signaling, such as PLC/DAG/IP3, PI3K/Akt, and Ras/MAPK-ERK pathways. BDNF can modulate the release of several neurotransmitters by interacting with other neuropeptides. Specifically, BDNF may regulate serotonin release (32), and modulates dopamine release via PLC-γ/IP3 pathway (33). Additionally, it may facilitate glutamate release through the MAPK/SynI activation (34), and controls GABAergic synaptic transmission influencing neuronal excitability. Reduction of BDNF levels affects formation of synaptic spine density, and decreases excitatory neurons leading to depression (35). BDNF can also interact with melanocortin, leptin, corticotrophin-releasing hormone (CRH), and thyrotropin-releasing hormone (TRH) playing critical physiopathological roles. BDNF and TrkB play a significant role in the neural circuitry triggering food intake and body weight, regulating appetite through the modulation of melanocortin signaling and hypothalamic serotonergic mechanisms. Pre-clinical data have shown that central alterations of BDNF signaling in mice results in severe hyperphagia and obesity (14, 36). On the other hand, high-fat diet promotes obesity, depressive-like phenotypes and reduction of BDNF in the hippocampus, a brain region important in controlling the mood. Moreover, hippocampal reductions of BDNF have been also reported in animal models of depression and in the postmortem brain samples of depressed patients (35). Interestingly, BDNF is potentially associated with obesity, depression and coronary syndromes (22). Being a versatile regulator of food intake as well as of inflammatory adipocyte maturation, it has been considered a critical mediator of obesity (14). In addition, compared to healthy subjects, reduced levels of BDNF have been found in patients with coronary syndromes (11, 12), and elevated BDNF appears to exert a protective effect (15). However, an open question remains as to whether BDNF has a fundamental role in CV complications, or it is just a bystander.

Waiting for the understanding of whether “came first the chicken or the egg,” several mechanisms have been proposed, starting from the knowledge that BDNF plays a cardio-protective role thanks to its antioxidant, pro-survivor and pro-angiogenic properties. It promotes the activation of reactive oxygen species detoxifying enzymes, increases cardiomyocyte survival under hypoxic conditions, stimulates new vessel assembly, and affects the structure, polymerization and viscoelastic properties of the fibrin clot, thus influencing the anticoagulation process (12, 37), without changing circulating fibrinogen levels (12, 29). According to these data (12, 29), in the SPHERE population no association between BDNF and fibrinogen levels was found. However, the ability of BDNF to modify fibrin clot structure suggests its clinical relevance as a potential biomarker of thrombosis including acute coronary syndrome (ACS). Indeed, as emerged from the PLATO study, fibrin clot properties independently predict adverse clinical outcomes after ACS (38). Future studies in depressed obese subjects should be performed to dissect out the potential relationship between BDNF and fibrin clot properties.

Interestingly, in a human study, Wu et al. found a positive correlation between severity of coronary artery stenosis and depression scores, and reported that lower serum BDNF levels were closely related to the development of depression in patients with stable coronary artery disease (39). Since it is well-established that reduced BDNF levels in the brain are linked to obesity, BDNF may represent the bridge among obesity, depression, and CV diseases. However, whether obesity itself may influence circulating BDNF levels is not well-defined as yet (22). In the present study, plasma BDNF levels did not correlate with BMI, whereas an evident relationship with BDI-II emerged, overall suggesting the strong impact of BDNF on depression. The lack of follow-up of the SPHERE study did not allow to assess whether antidepressant medications were able to raise plasma BDNF levels. We did not find any interaction between the antidepressant medications and BDNF levels (*p* = 0.9013) as well as between antidepressant medications and BDNF rs6265 (*p* = 0.0603) on BDI-II score.

Alterations in GABAergic, catecholaminergic and serotonergic systems as well as an aberrant cytokine production related to dysregulation of both innate and adaptive immune systems have been reported in people suffering from depression when compared to healthy individuals (40, 41). However, no significant correlations between catecholamine metabolites and BDNF in the blood of subjects with major depression have been reported (42), and platelet serotonin levels do not seem to go hand in hand with BDNF levels. Platelet serotonin levels do not differ between depressed and control subjects, whereas both reduced platelet BDNF content and plasma BDNF levels were found in subjects with depression (20, 30, 43). Then, it has been hypothesized that the altered BDNF levels observed in patients with depression are due to a reduced release from platelets which are considered one of the major circulation sources of BDNF. This latter is released by platelets upon their activation, raising to 100-fold higher levels in serum vs plasma (44) and correlating positively with platelets number (45). However, in people with depression, the reduction in serum and plasma BDNF levels seem to be independent of platelet reactivity (20), suggesting that other cells are involved in this modulation.

Recently, awareness has been raised regarding the relationship among obesity, depression and diet-related inflammation (46, 47). Gialluisi et al. (46) showed that the low-grade inflammation (INFLA)-score, based on four circulating inflammatory biomarkers (C-reactive protein, granulocyte-to-lymphocyte ratio, platelet and white blood cell counts), could explain the association between depressive symptoms and the dietary inflammatory index.

Considering that the studied cohort predominantly comprises overweight individuals, our findings are two fold: depression is directly related to a higher number of platelets, as in normal weight people (48); and the positive association between platelet number and depression score was mitigated by BDNF (Supplementary Figure 3). Altogether, this suggests that other cell populations (49, 50) could actively affect circulating BDNF levels. On this matter, we found that BDNF was positively correlated with white and red blood cells. This correlation can be explained, at least in part, by the ability of peripheral blood mononuclear (PBMC) to produce/secret BDNF (51, 52) that in turn can promote cell proliferation and survival. Indeed, BDNF induces the maturation, activation, proliferation and survival of T and B lymphocytes (49, 50). Since TrkB receptor was found in several bone marrow cells including osteoblasts, eosinophils, mastocytes, megakaryocytes and erythroblasts (49), it is possible to hypothesize that BDNF can affect also the behavior of these cell types.

Accumulating evidence shows that chronic inflammation is associated with depressive symptoms, and pre-clinical evidence supports the critical involvement of IFN-γ in the pathogenesis of this disease (53). In the SPHERE population, we did not measure levels of catecholamines, acetylcholine, serotonin, or of other neurotransmitters, but several cytokines have been evaluated. Interestingly, we did not find evidence of an association between BDI-II score and the measured cytokines, although IFN-γ affected the association BDNF-depression. The evidence that modulation of the BDNF-depression association occurs primarily at low IFN-γ levels suggests that under elevated inflammatory conditions, as found, e.g., in severe obesity (54), the ability of BDNF to impact on depressive symptoms is potentially negligible. *In vitro* data showed that T cells co-secreted larger amounts of BDNF and IFN-γ upon CD40 activation, whereas activated monocytes produced BDNF but not IFN-γ (55). Interestingly, obese patients displayed high levels of CD40 Ligand (56) suggesting that in this clinical condition activation of PBMC, rather than platelets, could be an important source of circulating BDNF.

The increased circulating levels of BDNF paralleled by the enhanced expression of the inflammatory markers MCP-1 and MIP1β, might be a consequence of ongoing pathological events. Abundant BDNF is present in the atherosclerotic lesions localizing in macrophages, vascular smooth muscle cells, as well as in perivascular adipose tissue (13, 37, 57). It may be secreted by activated monocytes in response to inflammatory cytokines (58) to exert anti-inflammatory and anti-apoptotic effects. However, when BDNF is chronically expressed in the presence of severe inflammatory conditions, it could have deleterious effects. BDNF enhances oxidative stress and promotes tissue factor (TF) activity in monocyte/macrophages (13, 51) and down regulates TrkB (57). Reduction in TrkB expression can contribute to atherosclerosis progression by affecting endothelial and smooth muscle cells survival and function, as well as heart development (37). Interestingly, our group showed that despite circulating levels of BDNF being lower in CV patients compared to healthy controls, a positive relationship between plasma BDNF and coronary plaque vulnerability, also including macrophage infiltration, appears to be present (11).

The relationship between the BDNFVal66Met polymorphism and depression is currently under debate. Specifically, the systematic review and meta-analyses performed by Kishi et al. (59) did not found any association between this polymorphism and depression, while noting that plasma BDNF levels were reduced in depressed patient. On the contrary, a positive association between this polymorphism and depression only in men, but not in women, was found by Verhagen (60). Similarly, the genetic association studies of the BDNFVal66Met polymorphism in obesity and cardiometabolic diseases provided inconsistent results. BDNF polymorphism could not differentiate carriers of Val vs. Met based on BMI (61, 62), or with CNS BDNF levels in suicidal depressed patients (63). However, this polymorphism may increase the risk of developing obesity when associated with the other polymorphisms related to the metabolic pathway (62). By contrast, other reports showed both a positive and an inverse association between obesity and the Met allele (64, 65). Finally, a greater adiposity based on BMI percentiles or BMI-for-age z-scores has been also described in Met allele carriers (61). In agreement with these studies, in the SPHERE cohort we did not find differences in BDNF allele frequency in relation with BMI, depression and circulating BDNF levels. Specific additional studies, including also subjects with a normal weight, may be needed in order to provide more definite conclusions.

Finally, by analyzing the EV-miRNA profiles we found that 139 miRNAs, known to target genes involved in atherothrombotic and CV processes, were associated with BDNF levels. Even if evidence on the ability of BDNF to directly modulate miRNAs expression is scanty, BDNF alters the expression of miR-433, miR-19b-1, miR-432, and miR-181c in human endothelial progenitor cells (66), and upregulates miR-214 in mouse embryonic stem cells (67). These findings provide evidence that some of the miRNAs here identified may be affected by BDNF.

Interestingly, the 139 miRNAs here identified target 10 genes (CYP2C19, F3, IL6, MTHFR, PLAT, PTGS2, SERPINE1, TFPI, THBD, TNF) present in all datasets analyzed. Remarkably, the protein-protein interaction analyses showed that BDNF could represent a critical hub among all these genes.

In addition, when data were analyzed focusing on depression state, 9 genes (ACE, APOE, BDNF, IL18, IL1B, IL6, MTHFR, MTR, and TNF) were shared with the atherothrombotic dataset. All these 9 genes were closely interlinked, and BDNF was directly related to 6 of them. Altogether these observations support the hypothesis that BDNF may be a common link among obesity, CV diseases and depression.

Remarkably, ACE has been identified as the target gene for both atherothrombotic complications (68) and management of depression (69). Overexpression of ACE2 in a transgenic animal model attenuated plaque vulnerability and inflammation (70) and reduced the propensity to develop an anxiety phenotype (29). Recently, it has been demonstrated that down regulation of ACE2 by SARS-CoV-2 exacerbates the thrombotic COVID-19 severity and predisposes to depression (71). Changes in the levels of serum BDNF were observed in post-infected-COVID-19 symptomatic patients (72), predicting an intensive care admission and a worse prognosis (73).

In this scenario, Lorkiewicz et al. (74) proposed to include BDNF among biomarkers of COVID-19-derived depression, whereas De Sousa et al. (75) underlined how the rise of BDNF levels, induced by physical activity, was crucial for maintaining good mental health after COVID-19 syndrome.

Of note, alterations in the ACE signaling-pathway affected the release of modulators of fibrinolytic processes, such as Serpin1 and Tissue-type plasminogen activator (PLAT). Although Serpin1 and PLAT were not co-shared between the depression and atherothrombotic phenotypes, it has been hypothesized that PLAT and Serpin1, by modulating the proteolytic ability of plasmin, may regulate the cleavage of pro-BDNF precursor to the mature form (76). All these findings clearly summarize the strong connections of these genes in obesity, depression, and CV disease.

Our analysis also identified the possible involvement of prostaglandin-endoperoxide synthase 2 (PTGS2), a key enzyme in the production of prostanoids. Increased PTGS2 levels have been detected in atherosclerotic plaques (77) as well as in the rat brain showing depression-like phenotypes (78). The imbalance in PTGS2-BDNF signaling pathway has been suggested to be one of the pathogenetic mechanisms of depression (79).

Although P-selectin, thromboglobulin, and MTHFR, a key modulator of purine and thymidylate biosynthesis, play a critical role in thrombosis (80, 81), and their alterations have been observed in subjects with depression (82, 83), definitive potential relationship with BDNF is actually missing.

The present study presents some limitations. Firstly, this is a monocentric study, and the questionnaire was self-administered, without final psychiatric interview diagnosing the depressive state. Moreover, the BDI-II questionnaire was administered only once at the recruitment of patients. BDI-II has been widely applied including earlier studies in cardiac patients (84); having been successfully administered both to adolescents and adults, as a tool of both medical assistance and research, it represents an established self-rating scale. A large portion of subjects classified with depression has subclinical or mild symptomatology, only a fraction of those taking psychotropic medications. Although we collected extensive information on habitual physical activity and education, many other factors may influence BDNF levels, and not all potential confounders could be taken into consideration here. The follow-up of these subjects is not available at present, limiting the potential prognostic value of BDNF for cardiovascular events at this moment. No other traditional markers of predisposition to thrombosis have been measured. Finally, the molecular mechanisms underlying the relation between BDNF and the emerged miRNAs affecting obesity, depression and CVD have not been investigated as yet.

## Conclusions

The described link among BDNF and obesity, classical and non-classical CV risk factors in individuals with obesity and different degrees of depression provide one more explanatory variable (i.e., BDNF) as a culprit. In the present report, there was clear evidence of a negative association between BDNF and major clinical depression scores, with no impact of BDNF genetic polymorphisms. Although the association between BDNF and depression risk factors was lost in the presence of elevated levels of a major inflammatory marker (IFN-γ), the detection of this link is novel as was the association with miRNAs related to atherothrombosis and depression. This last analysis detected a large number of up-regulated and down-regulated genes, eventually allowing to create a network analysis of potential use in the molecular characterization of both CV and depression in obese individuals.

## Data Availability Statement

The original contributions presented in the study are included in the article/Supplementary Material, further inquiries can be directed to the corresponding authors.

## Ethics Statement

The studies involving human participants were reviewed and approved by Ethics Committee of Fondazione IRCCS Cà Granda OspedaleMaggiore Policlinico (approval 1425).

## Author Contributions

PA, CM, MZ, and MG performed the experiments. CF performed the statistical analyses. LD isolated the EV-derived miRNAs. GS performed the bioinformatic analyses of miRNAs. LV was responsible for the recruitment of SPHERE cohort. MB was responsible for the evaluation of BDI-II score. LS, AI, AP, and CS critically reviewed the manuscript. SB, MR, and VB conceived the study and wrote the first draft of the manuscript. All authors contributed to the article and approved the submitted version.

## Funding

This work was partially supported by Fondazione Cariplo (2018-0511 to MR and 2018-0525 to SB), Banca di Credito Cooperativa di Milano (donation to MR), European Research Council (ERC-2011-StG 282,413 to VB), and Italian Ministry of Health, Rome, Italy (Ricerca Corrente RC 2021, and 5 × 1000 2018).

## Conflict of Interest

The authors declare that the research was conducted in the absence of any commercial or financial relationships that could be construed as a potential conflict of interest.

## Publisher's Note

All claims expressed in this article are solely those of the authors and do not necessarily represent those of their affiliated organizations, or those of the publisher, the editors and the reviewers. Any product that may be evaluated in this article, or claim that may be made by its manufacturer, is not guaranteed or endorsed by the publisher.
