# Supplementary material for: Brain-Derived Neurotrophic Factor and Extracellular Vesicle-Derived miRNAs in an Italian Cohort of Individuals With Obesity: A Key to Explain the Link Between Depression and Atherothrombosis
Source: Front Cardiovasc Med. 2022 Jul 13;9:906483. doi: 10.3389/fcvm.2022.906483 (PMC9326054; doi:10.3389/fcvm.2022.906483)
Supplement: Supplementary file 1 [file Data_Sheet_1.PDF]

Supplementary Material

Supplementary Table 1. Concentration of cytokines (pg/ml) measured in 739 participants.

Cytokines	Abbreviations	Number of subjects with citokine < LLOQ	
Interferon-γ	IFN-γ	161 (21.8%)	10.4 ± 5.4
Interleukin-8	IL-8	0 (0.0%)	9.7 ± 9.9
Interleukin-10	IL-10	102 (13.8%)	4.2 [3.1;5.6]
Interleukin-18	IL-18	0 (0.0%)	276.5 [208.5;356]
Macrophage inflammatory protein-1α (CCL3)	MIP-1α	76 (10.3%)	87 [58.5;120]
Macrophage inflammatory protein-1β (CCL4)	MIP-1β	0 (0.0%)	186.5 [147;234]
Monocyte chemoattractant protein-1 (CCL2)	MCP-1	1 (0.1%)	174 [142;215.5]
Tumor necrosis factor-α (TNF)	TNF-α	213 (28.8%)	5.7 [3.2;8.5]

LLOQ indicates Lower Limit of Quantitation is the lowest concentration of an analyte in a sample that can be reliably detected and at which the total error meets the laboratory's requirements for accuracy. Variables are expressed as mean ± standard deviation (SD) or as median [first quartile-third quartile] if not normally distributed.

Supplementary Table 2. Slope coefficients from univariate negative binomial regression models to evaluate association between BDI-II score and demographics and clinical characteristics of participants.

OUTCOME: BDI-II SCORE

**Independent variable**	**β**	**SE**	**95% CI**		**P-value**	
BDNF, pg/mL	-0.009	0.005	-0.019	0.001	0.0870	
**BDNF VAL66**						
**Val/Val**	**REF**	**-**	**-**		**-**	0.1915
**Val/Met**	0.110	0.061	-0.009	0.229	0.0700	
**Met/Met**	0.048	0.127	-0.202	0.297	0.7086	
Age, years	0.001	0.002	-0.003	0.005	0.7501	
**Gender**						
**Females**	**0.557**	**0.063**	**0.434**	**0.680**	**<0.0001**	
**Males**	**REF**	**-**	**-**	**-**		
**BMI, Kg/m^2**	**0.015**	**0.005**	**0.005**	**0.025**	**0.0032**	
TC, mg/dL	-0.0003	0.0007	-0.0016	0.0011	0.6921	
HDL-C, mg/dL	0.0002	0.0019	-0.0035	0.0038	0.9361	
LDL-C, mg/dL	-0.0003	0.0008	-0.0018	0.0012	0.7359	
non-HDL-C, mg/dL	-0.0003	0.0007	-0.0016	0.0010	0.6726	
LDL-C/HDL-C	-0.0047	0.0303	-0.0642	0.0547	0.8763	
TC/HDL-C	-0.0084	0.0249	-0.0571	0.0404	0.7364	
TG, mg/dL	0.0003	0.0004	-0.0005	0.0011	0.4294	
CRP, mg/L	0.0667	0.0407	-0.0130	0.1464	0.1011	
Serum creatinine, mg/dL	-0.0773	0.0723	-0.2190	0.0644	0.2847	
Uric acid, mg/dL	-0.0340	0.0205	-0.0742	0.0061	0.0969	
**Fibrinogen, mg/d**	**0.0010**	**0.0005**	**0.0000**	**0.0019**	**0.0418**	
AST, U/L	-0.0025	0.0032	-0.0087	0.0036	0.4195	
ALT, U/L	-0.0007	0.0014	-0.0035	0.0021	0.6206	
GGT, U/L	-0.0001	0.0012	-0.0024	0.0023	0.9449	
Homocysteine, µmol/L	-0.0115	0.0061	-0.0235	0.0006	0.0625	
TSH, U/mL	-0.0178	0.0217	-0.0604	0.0248	0.4140	
Glucose, mg/dL	0.0019	0.0011	-0.0003	0.0041	0.0882	
**HbA1C, mmol/mol**	**0.0089**	**0.0033**	**0.0024**	**0.0153**	**0.0075**	
Insulin level, U/mL	0.0054	0.0030	-0.0004	0.0113	0.0702	
**HOMA-IR score**	**0.0207**	**0.0102**	**0.0008**	**0.0407**	**0.0417**	
**QUICKI score**	**-5.3912**	**2.1260**	**-9.5582**	**-1.2243**	**0.0112**	
**PCSK9, ng/mL**	**0.0012**	**0.0003**	**0.0006**	**0.0018**	**<0.0001**	
BDNF, pg/mL	-0.009	0.005	-0.019	0.001	0.0870	
Cytokines						
IFN-γ, pg/mL	-0.0065	0.0044	-0.0151	0.0021	0.1383	
IL-8, pg/mL	0.0046	0.0030	-0.0014	0.0105	0.1334	
IL-10, pg/mL	0.0011	0.0009	-0.0006	0.0029	0.1983	
IL-18, pg/mL	0.0001	0.0002	-0.0003	0.0004	0.6408	

MIP-1α, pg/mL	-0.0007	0.0005	-0.0017	0.0003	0.1710	
MIP-1β, pg/mL	0.0002	0.0003	-0.0003	0.0007	0.4444	
MCP-1, pg/mL	0.0001	0.0002	-0.0004	0.0006	0.6958	
TNF-α, pg/mL	0.0018	0.0014	-0.0009	0.0046	0.1969	
Blood count						
White blood cells (x10^3/µL)	0.0255	0.0166	-0.0071	0.0581	0.1247	
**Red blood cells** (x10^6/µL)	**-0.2520**	**0.0605**	**-0.3706**	**-0.1334**	**<0.0001**	
**Haemoglobin (g/dL)**	**-0.1421**	**0.0220**	**-0.1851**	**-0.0990**	**<0.0001**	
**Haematocrit (%)**	**-0.0480**	**0.0087**	**-0.0651**	**-0.0310**	**<0.0001**	
Mean Corpuscolar Volume (fL)	-0.0029	0.0044	-0.0116	0.0058	0.5179	
**Platelets (x10^3/µL)**	**0.0016**	**0.0005**	**0.0007**	**0.0025**	**0.0003**	
Smoking status						
Former smoker	0.026	0.062	-0.096	0.148	0.6782	0.9174
Current smoker	0.010	0.080	-0.146	0.166	0.9020	
Never smoker	REF	-	-	-	-	
**Employment status**						
**Unemployed**	**0.300**	**0.101**	**0.102**	**0.498**	**0.0030**	**0.0114**
**Pensioner**	**0.050**	**0.070**	**-0.086**	**0.187**	**0.4718**	
**Housewife**	**0.180**	**0.103**	**-0.022**	**0.382**	**0.0813**	
**Employee**	**REF**	**-**	**-**	**-**	**-**	
**Education**						
**Secondary school**	**-0.022**	**0.111**	**-0.239**	**0.1949**	**0.8431**	**0.0191**
**High school**	**-0.216**	**0.107**	**-0.426**	**-0.0056**	**0.0442**	
**University or more**	**-0.164**	**0.113**	**-0.386**	**0.0582**	**0.1479**	
**Primary school or less**	**REF**	**-**	**-**	**-**	**-**	
**Lifestyle**						
**Active**	**-0.175**	**0.061**	**-0.295**	**-0.055**	**0.004**	**0.0065**
**Sporty**	**-0.225**	**0.117**	**-0.454**	**0.004**	**0.055**	
**Sedentary**	**REF**	**-**	**-**	**-**	**-**	
**Heart rate, bpm**	0.0043	0.0028	-0.0012	0.0098	0.1270	
**Blood pressure, mmHg**						
**Sistolic**	**-0.005**	**0.002**	**-0.008**	**-0.001**	**0.0078**	
**Diastolic**	**-0.008**	**0.003**	**-0.013**	**-0.002**	**0.0085**	
**MAP**	**-0.007**	**0.003**	**-0.013**	**-0.002**	**0.0038**	
**Diabetes**						
**Yes**	**0.215**	**0.090**	**0.039**	**0.392**	**0.0168**	**0.0198**
**Pre-diabetes**	**-0.041**	**0.061**	**-0.160**	**0.079**	**0.5022**	
**No**	**REF**	**-**	**-**	**-**		
**Use of antidepressant medications**						
**Yes**	**0.418**	**0.081**	**0.260**	**0.576**	**<0.0001**	
**No**	**REF**	**-**	**-**	**-**		
Antihypertensive medications						
Yes	0.0327	0.0581	-0.0813	0.1466	0.5741	
No	REF	-	-	-		

Statin medications							
	Yes	0.137	0.092	-0.044	0.318	0.1381	
	No	REF	-	-	-		
**Diabetes medications**							
	**Yes**	**0.306**	**0.104**	**0.102**	**0.509**	**0.0032**	**0.0025**
	**No**	**REF**	**-**	**-**	**-**		

Abbreviations: ALT: alanine aminotransferase; AST: Aspartate aminotransferase; BDI: Beck Depression Inventory; BDNF: brain-derived neurotrophic factor; BMI: body mass index; CRP: C-reactive proteins; GGT: gamma-glutamyltransferase; HbA1C: glycated haemoglobin; HDL-C: high-density lipoprotein cholesterol; HOMA-IR: omeostasis model assessment of insulin resistance; IFN-γ: interferon gamma; IL: interleukin; MCP-1: monocyte chemoattractant protein-1; MIP-1α: macrophage inflammatory protein-1 alpha; MIP-1β: macrophage inflammatory protein-1 beta; LDL-C: low-density lipoprotein cholesterol; MAP: mean arterial pressure; PCSK9: proprotein convertase subtilisin/kexin type 9; QUICKI: quantitative insulin sensitivity check index; TC: total cholesterol; TG: triglyceride; TSH: thyroid stimulating hormone; WC: waist circumference.

Supplementary Table 3. EVs-derived miRNAs expressed in at least one subject

Quant StudioAssay ID	Assay Name	miRBase ID (v21) or NCBI Name (for Controls)	miRBase Alias	mirna_name	Effect	Δ%	95% CI		P-value	FDR pvalue
001026	hsa-miR-432	hsa-miR-432-5p,	hsa-miR-432(17),	miR_432_001026	bdnf	6.8955	3.8429	10.0379	<0.0001	**0.0010**
002098	hsa-miR-223#	hsa-miR-223-5p	hsa-miR-223*(17)	miR_223__002098	bdnf	6.8879	4.0074	9.8480	<0.0001	**0.0010**
002301	hsa-miR-22#	hsa-miR-22-5p,	hsa-miR-22*(17),	miR_22__002301	bdnf	6.9339	3.9008	10.0555	<0.0001	**0.0010**
002304	hsa-miR-199a-3p	hsa-miR-199a-3p, hsa-miR-199b-3p,		miR_199a_3p_002304	bdnf	7.6044	4.2534	11.0632	<0.0001	**0.0010**
001186	hsa-miR-134	hsa-miR-134-5p,	hsa-miR-134(19),	miR_134_001186	bdnf	7.0565	3.7156	10.5049	<0.0001	**0.0024**
001663	hsa-miR-495	hsa-miR-495-3p,	hsa-miR-495(18),	miR_495_001663	bdnf	9.1085	4.8152	13.5777	<0.0001	**0.0024**
002446	hsa-miR-28-3p	hsa-miR-28-3p,	rno-miR-28*(18)	miR_28_3p_002446	bdnf	7.8977	4.1034	11.8302	<0.0001	**0.0026**
000557	hsa-miR-369-3p	hsa-miR-369-3p,	hsa-miR-369(6)	miR_369_3p_000557	bdnf	6.6381	3.3503	10.0305	0.0001	**0.0041**
000402	hsa-miR-24	hsa-miR-24-3p,	hsa-miR-24(17),	miR_24_000402	bdnf	4.2009	2.0794	6.3665	0.0001	**0.0043**
001286	hsa-miR-539	hsa-miR-539-5p,	hsa-miR-539(17),	miR_539_001286	bdnf	6.5888	3.2437	10.0423	0.0001	**0.0043**
002295	hsa-miR-223	hsa-miR-223-3p,	hsa-miR-223(17),	miR_223_002295	bdnf	3.6945	1.8497	5.5728	0.0001	**0.0043**
002349	hsa-miR-574-3p	hsa-miR-574-3p,	NA	miR_574_3p_002349	bdnf	6.1894	3.0391	9.4360	0.0001	**0.0043**
002202	hsa-miR-889	hsa-miR-889-3p,	hsa-miR-889(19),	miR_889_002202	bdnf	6.0076	2.8681	9.2429	0.0002	**0.0053**
002299	hsa-miR-191	hsa-miR-191-5p,	hsa-miR-191(17),	miR_191_002299	bdnf	4.3728	2.0949	6.7015	0.0002	**0.0053**
002305	hsa-miR-30d#	hsa-miR-30d-3p,	hsa-miR-30d*(17),	miR_30d__002305	bdnf	4.3607	2.1083	6.6628	0.0001	**0.0053**
002139	hsa-miR-93#	hsa-miR-93-3p,	hsa-miR-93*(17),	miR_93__002139	bdnf	5.4095	2.5163	8.3843	0.0002	**0.0063**
002376	hsa-miR-543	hsa-miR-543,		miR_543_002376	bdnf	5.1391	2.4061	7.9452	0.0002	**0.0063**
002854	hsa-miR-1243	hsa-miR-1243	NA	miR_1243_002854	bdnf	-28.0612	-39.5429	-14.3990	0.0002	**0.0063**
002277	hsa-miR-320	hsa-miR-320a,	hsa-miR-320(10.1),	miR_320_002277	bdnf	4.7127	2.1773	7.3110	0.0002	**0.0066**
002325	hsa-miR-744#	hsa-miR-744-3p,	hsa-miR-744*(17),	miR_744__002325	bdnf	4.7715	2.1921	7.4161	0.0003	**0.0067**
002365	hsa-miR-494	hsa-miR-494-3p,	hsa-miR-494(19),	miR_494_002365	bdnf	5.9045	2.6751	9.2355	0.0003	**0.0073**
002322	hsa-miR-671-3p	hsa-miR-671-3p,	NA	miR_671_3p_002322	bdnf	4.3997	1.9618	6.8958	0.0004	**0.0083**
002332	hsa-miR-409-3p	hsa-miR-409-3p,	NA	miR_409_3p_002332	bdnf	8.1826	3.6134	12.9533	0.0004	**0.0083**
000509	hsa-miR-205	hsa-miR-205-5p,	hsa-miR-205(17),	miR_205_000509	bdnf	-3.6604	-5.6261	-1.6537	0.0004	**0.0088**
002863	hsa-miR-1290	hsa-miR-1290,	NA	miR_1290_002863	bdnf	-2.4192	-3.7369	-1.0835	0.0004	**0.0089**
000454	hsa-miR-130a	hsa-miR-130a-3p,	hsa-miR-130a(17),	miR_130a_000454	bdnf	5.0896	2.1948	8.0663	0.0005	**0.0099**
001285	hsa-miR-487b	hsa-miR-487b-3p,	hsa-miR-487b(19),	miR_487b_001285	bdnf	5.7043	2.4443	9.0680	0.0005	**0.0099**
002642	hsa-miR-151-5P	hsa-miR-151a-5p,	hsa-miR-151-5p(17),	miR_151_5P_002642	bdnf	5.4053	2.3203	8.5833	0.0005	**0.0099**
002174	hsa-miR-27b#	hsa-miR-27b-5p,	hsa-miR-27b*(17),	miR_27b__002174	bdnf	3.4929	1.4928	5.5325	0.0006	**0.0101**
002184	hsa-miR-339-3p	hsa-miR-339-3p,	NA	miR_339_3p_002184	bdnf	5.1835	2.2081	8.2456	0.0006	**0.0101**
002156	hsa-miR-337-5p	hsa-miR-337-5p,	NA	miR_337_5p_002156	bdnf	4.5613	1.9258	7.2649	0.0006	**0.0106**
000475	hsa-miR-152	hsa-miR-152-3p,	hsa-miR-152(19),	miR_152_000475	bdnf	4.5030	1.8862	7.1870	0.0007	**0.0111**

002227	hsa-miR-323-3p	hsa-miR-323a-3p,	hsa-miR-323-3p(17),	miR_323_3p_002227	bdnf	5.1183	2.1299	8.1942	0.0007	**0.0112**
002434	hsa-miR-628-3p	hsa-miR-628-3p,	NA	miR_628_3p_002434	bdnf	3.3280	1.3762	5.3175	0.0008	**0.0120**
002259	hsa-miR-340#	hsa-miR-340-3p,	hsa-miR-340*(17)	miR_340__002259	bdnf	5.1619	2.1114	8.3036	0.0008	**0.0122**
000511	hsa-miR-208	hsa-miR-208a-3p,	hsa-miR-208(10),hsa-miR-208a(19),	miR_208_000511	bdnf	-1.9398	-3.0597	-0.8070	0.0009	**0.0122**
001101	hsa-miR-329	hsa-miR-329-3p,	hsa-miR-329(19),	miR_329_001101	bdnf	3.4398	1.3912	5.5297	0.0010	**0.0132**
000524	hsa-miR-221	hsa-miR-221-3p,	hsa-miR-221(17),	miR_221_000524	bdnf	5.4025	2.1535	8.7548	0.0010	**0.0135**
001998	hsa-miR-769-5p	hsa-miR-769-5p	NA	miR_769_5p_001998	bdnf	4.1475	1.6574	6.6987	0.0010	**0.0135**
001150	hsa-miR-516b	hsa-miR-516b-5p,	hsa-miR-516-5p(7),hsa-miR-516b(17),	miR_516b_001150	bdnf	-1.8056	-2.8707	-0.7289	0.0011	**0.0140**
000544	hsa-miR-330	hsa-miR-330-3p,	hsa-miR-330(9.2)	miR_330_000544	bdnf	7.7349	3.0107	12.6758	0.0012	**0.0145**
001567	hsa-miR-604	hsa-miR-604	NA	miR_604_001567	bdnf	-1.7486	-2.7907	-0.6954	0.0012	**0.0149**
000531	hsa-miR-302b	hsa-miR-302b-3p,	hsa-miR-302b(17),	miR_302b_000531	bdnf	-1.8628	-2.9823	-0.7304	0.0014	**0.0153**
001986	hsa-miR-766	hsa-miR-766-3p,	hsa-miR-766(17)	miR_766_001986	bdnf	4.6430	1.7820	7.5845	0.0014	**0.0153**
002147	hsa-miR-342-5p	hsa-miR-342-5p,	NA	miR_342_5p_002147	bdnf	-1.7244	-2.7606	-0.6772	0.0013	**0.0153**
002392	hsa-miR-301b	hsa-miR-301b-3p,	hsa-miR-301b(20)	miR_301b_002392	bdnf	4.6553	1.7890	7.6023	0.0014	**0.0153**
000542	hsa-miR-326	hsa-miR-326,	NA	miR_326_000542	bdnf	4.8519	1.8319	7.9614	0.0015	**0.0159**
000546	hsa-miR-335	hsa-miR-335-5p,	hsa-miR-335(17),	miR_335_000546	bdnf	5.8709	2.2131	9.6597	0.0015	**0.0159**
002351	hsa-miR-576-3p	hsa-miR-576-3p,	NA	miR_576_3p_002351	bdnf	4.4011	1.6729	7.2025	0.0015	**0.0159**
001988	hsa-miR-598	hsa-miR-598-3p,	hsa-miR-598(19),	miR_598_001988	bdnf	4.9876	1.8724	8.1979	0.0016	**0.0162**
001603	hsa-miR-650	hsa-miR-650,	NA	miR_650_001603	bdnf	-1.6453	-2.6545	-0.6257	0.0017	**0.0163**
002619	hsa-let-7b	hsa-let-7b-5p,	hsa-let-7b(17),	let_7b_002619	bdnf	-4.1614	-6.6604	-1.5956	0.0017	**0.0163**
000566	hsa-miR-377	hsa-miR-377-3p,	hsa-miR-377(17),	miR_377_000566	bdnf	-1.6078	-2.5960	-0.6095	0.0017	**0.0163**
000600	hsa-miR-299-5p	hsa-miR-299-5p,	hsa-miR-299(6),	miR_299_5p_000600	bdnf	-2.1249	-3.4412	-0.7906	0.0019	**0.0181**
002093	hsa-miR-486-3p	hsa-miR-486-3p,		miR_486_3p_002093	bdnf	-1.8913	-3.0668	-0.7015	0.0020	**0.0181**
002431	hsa-miR-625	hsa-miR-625-5p	hsa-miR-625(17)	miR_625_002431	bdnf	4.9759	1.7852	8.2665	0.0021	**0.0191**
001984	hsa-miR-590-5p	hsa-miR-590-5p,	hsa-miR-590(9.2)	miR_590_5p_001984	bdnf	4.0219	1.4412	6.6683	0.0022	**0.0192**
002406	hsa-let-7e	hsa-let-7e-5p,	hsa-let-7e(17),	let_7e_002406	bdnf	7.2990	2.5739	12.2418	0.0022	**0.0196**
002198	hsa-miR-125a-5p	hsa-miR-125a-5p,	NA	miR_125a_5p_002198	bdnf	5.7338	2.0149	9.5882	0.0023	**0.0201**
000408	hsa-miR-27a	hsa-miR-27a-3p,	hsa-miR-27a(17),	miR_27a_000408	bdnf	4.3321	1.5104	7.2322	0.0025	**0.0202**
002169	hsa-miR-106a	hsa-miR-106a-5p,	hsa-miR-106a(17),	miR_106a_002169	bdnf	3.2109	1.1286	5.3362	0.0024	**0.0202**
002303	hsa-miR-450a	hsa-miR-450a-5p,	hsa-miR-450a(17),	miR_450a_002303	bdnf	-1.6847	-2.7595	-0.5981	0.0025	**0.0202**
002324	hsa-miR-744	hsa-miR-744-5p,	hsa-miR-744(17),	miR_744_002324	bdnf	4.7832	1.6600	8.0024	0.0025	**0.0202**
002340	hsa-miR-423-5p	hsa-miR-423-5p,	NA	miR_423_5p_002340	bdnf	4.4353	1.5456	7.4072	0.0025	**0.0202**
002102	hsa-miR-34b	hsa-miR-34b-3p,	hsa-miR-34b(17)	miR_34b_002102	bdnf	-1.7503	-2.8786	-0.6089	0.0028	**0.0219**
000592	hsa-miR-136	hsa-miR-136-5p,	hsa-miR-136(17),	miR_136_000592	bdnf	-10.0717	-16.1467	-3.5567	0.0030	**0.0228**
001585	hsa-miR-641	hsa-miR-641	NA	miR_641_001585	bdnf	-1.5052	-2.4861	-0.5144	0.0031	**0.0228**
				miR_1233_002768	bdnf	-1.6093	-2.6569	-0.5504	0.0030	**0.0228**

000396	hsa-miR-19b	hsa-miR-19b-3p,	hsa-miR-19b(17),	miR_19b_000396	bdnf	2.7365	0.9092	4.5970	0.0033	**0.0234**
000569	hsa-miR-380-3p	hsa-miR-380-3p,	hsa-miR-380(17)	miR_380_3p_000569	bdnf	-1.4966	-2.4790	-0.5043	0.0033	**0.0234**
002676	hsa-miR-144	hsa-miR-144-3p,	hsa-miR-144(17),	miR_144_002676	bdnf	-1.7090	-2.8274	-0.5777	0.0032	**0.0234**
002255	hsa-miR-149	hsa-miR-149-5p,	hsa-miR-149(17),	miR_149_002255	bdnf	-1.5289	-2.5339	-0.5134	0.0033	**0.0234**
002902	hsa-miR-1300	NA	hsa-miR-1300(13),	miR_1300_002902	bdnf	-1.5526	-2.5757	-0.5186	0.0034	**0.0237**
000478	hsa-miR-154#	hsa-miR-154-3p,	hsa-miR-154*(17),	miR_154__000478	bdnf	3.0822	1.0085	5.1985	0.0035	**0.0240**
000468	hsa-miR-146a	hsa-miR-146a-5p,	hsa-miR-146(6),hsa-miR-146a(17),	miR_146a_000468	bdnf	5.6793	1.7964	9.7103	0.0039	**0.0240**
000539	hsa-miR-324-5p	hsa-miR-324-5p,	NA	miR_324_5p_000539	bdnf	5.0642	1.6143	8.6312	0.0038	**0.0240**
001531	hsa-miR-564	hsa-miR-564,	NA	miR_564_001531	bdnf	-1.4710	-2.4547	-0.4773	0.0039	**0.0240**
001557	hsa-miR-624	hsa-miR-624-5p	hsa-miR-624(9.2),hsa-miR-624*(17)	miR_624_001557	bdnf	3.7062	1.1993	6.2751	0.0036	**0.0240**
001584	hsa-miR-640	hsa-miR-640,	NA	miR_640_001584	bdnf	-1.4723	-2.4633	-0.4711	0.0041	**0.0240**
001610	hsa-miR-411	hsa-miR-411-5p,	hsa-miR-411(17),	miR_411_001610	bdnf	5.8918	1.8766	10.0652	0.0038	**0.0240**
002160	hsa-miR-148b#	hsa-miR-148b-5p	hsa-miR-148b*(17)	miR_148b__002160	bdnf	3.6792	1.1815	6.2385	0.0038	**0.0240**
002203	hsa-miR-875-5p	hsa-miR-875-5p,	NA	miR_875_5p_002203	bdnf	-1.5208	-2.5401	-0.4908	0.0040	**0.0240**
				miR_526b_002382	bdnf	-1.4638	-2.4469	-0.4708	0.0040	**0.0240**
002437	hsa-miR-20a#	hsa-miR-20a-3p	hsa-miR-20a*(17)	miR_20a__002437	bdnf	3.9963	1.2577	6.8090	0.0041	**0.0240**
002841	hsa-miR-1183	hsa-miR-1183,	NA	miR_1183_002841	bdnf	-2.4739	-4.1008	-0.8193	0.0036	**0.0240**
002867	hsa-miR-1305	hsa-miR-1305	NA	miR_1305_002867	bdnf	-1.4918	-2.4850	-0.4885	0.0037	**0.0240**
002893	hsa-miR-1247	hsa-miR-1247-5p,	hsa-miR-1247(17),	miR_1247_002893	bdnf	-1.4701	-2.4584	-0.4717	0.0041	**0.0240**
000439	hsa-miR-103	hsa-miR-103a-3p,	hsa-miR-103(16),hsa-miR-103a(17),	miR_103_000439	bdnf	4.5931	1.3931	7.8941	0.0047	**0.0243**
000456	hsa-miR-130b	hsa-miR-130b-3p,	hsa-miR-130b(17),	miR_130b_000456	bdnf	4.8064	1.4931	8.2278	0.0043	**0.0243**
001553	hsa-miR-622	hsa-miR-622,	NA	miR_622_001553	bdnf	-2.8142	-4.7096	-0.8811	0.0046	**0.0243**
002099	hsa-miR-224	hsa-miR-224-5p	hsa-miR-224(17)	miR_224_002099	bdnf	4.9060	1.4932	8.4336	0.0046	**0.0243**
002141	hsa-miR-99a#	hsa-miR-99a-3p	hsa-miR-99a*(17)	miR_99a__002141	bdnf	-1.4365	-2.4196	-0.4435	0.0048	**0.0243**
002261	hsa-miR-135b	hsa-miR-135b-5p,	hsa-miR-135b(17),	miR_135b_002261	bdnf	-2.8555	-4.7868	-0.8851	0.0048	**0.0243**
002350	hsa-miR-576-5p	hsa-miR-576-5p	NA	miR_576_5p_002350	bdnf	-1.4646	-2.4548	-0.4645	0.0043	**0.0243**
002378	hsa-miR-125b-1#	hsa-miR-125b-1-3p,	hsa-miR-125b-1*(17),	miR_125b_1__002378	bdnf	-3.3617	-5.5994	-1.0709	0.0043	**0.0243**
002403	hsa-miR-519d	hsa-miR-519d-3p,	hsa-miR-519d(19)	miR_519d_002403	bdnf	-1.4423	-2.4249	-0.4498	0.0046	**0.0243**
002415	hsa-miR-519a	hsa-miR-519a-3p,	hsa-miR-519a(17)	miR_519a_002415	bdnf	-1.4361	-2.4185	-0.4437	0.0047	**0.0243**
002435	hsa-miR-501-3p	hsa-miR-501-3p,	NA	miR_501_3p_002435	bdnf	-2.2531	-3.7800	-0.7019	0.0046	**0.0243**
002444	hsa-miR-26b#	hsa-miR-26b-3p,	hsa-miR-26b*(17),	miR_26b__002444	bdnf	3.4041	1.0330	5.8308	0.0048	**0.0243**
002827	hsa-miR-1301	hsa-miR-1301-3p,	hsa-miR-1301(19)	miR_1301_002827	bdnf	-1.4448	-2.4334	-0.4462	0.0048	**0.0243**
000428	hsa-miR-34c	hsa-miR-34c-5p,	hsa-miR-34c(9.2),	miR_34c_000428	bdnf	-6.7283	-11.1391	-2.0985	0.0049	**0.0247**
000540	hsa-miR-325	hsa-miR-325,	NA	miR_325_000540	bdnf	-1.5248	-2.5753	-0.4630	0.0051	**0.0250**
001138	hsa-miR-379	hsa-miR-379-5p,	hsa-miR-379(17),	miR_379_001138	bdnf	4.8193	1.4209	8.3316	0.0052	**0.0250**
001149	hsa-miR-516-3p			miR_516_3p_001149	bdnf	-1.4699	-2.4859	-0.4434	0.0052	**0.0250**
002258	hsa-miR-340	hsa-miR-340-5p,	hsa-miR-340(17),	miR_340_002258	bdnf	4.7365	1.3899	8.1936	0.0053	**0.0250**

002314	hsa-miR-7-2#	hsa-miR-7-2-3p	hsa-miR-7-2*(17)	miR_7_2__002314	bdnf	-1.4187	-2.4012	-0.4262	0.0053	**0.0250**
002364	hsa-miR-493	hsa-miR-493-3p,	hsa-miR-493(17),	miR_493_002364	bdnf	2.7319	0.8177	4.6825	0.0051	**0.0250**
002908	hsa-miR-1296	hsa-miR-1296-5p,	hsa-miR-1296(19)	miR_1296_002908	bdnf	-1.4338	-2.4243	-0.4332	0.0052	**0.0250**
001182	hsa-miR-124a	hsa-miR-124-3p,	hsa-miR-124(17),	miR_124a_001182	bdnf	-1.5659	-2.6578	-0.4618	0.0056	**0.0259**
002159	hsa-miR-135b#	hsa-miR-135b-3p,	hsa-miR-135b*(17),	miR_135b__002159	bdnf	-1.8972	-3.2144	-0.5620	0.0056	**0.0259**
002283	hsa-let-7d	hsa-let-7d-5p,	hsa-let-7d(17),	let_7d_002283	bdnf	4.8958	1.4108	8.5007	0.0057	**0.0259**
002758	hsa-miR-1226#	hsa-miR-1226-5p	hsa-miR-1226*(17)	miR_1226__002758	bdnf	-1.5123	-2.5775	-0.4355	0.0061	**0.0277**
002100	hsa-miR-136#	hsa-miR-136-3p,	hsa-miR-136*(17),	miR_136__002100	bdnf	4.6324	1.2979	8.0767	0.0062	**0.0278**
002188	hsa-miR-943	hsa-miR-943,	NA	miR_943_002188	bdnf	-1.4113	-2.4075	-0.4049	0.0062	**0.0278**
002122	hsa-miR-376c	hsa-miR-376c-3p,	hsa-miR-376c(18),	miR_376c_002122	bdnf	5.0713	1.4139	8.8606	0.0063	**0.0278**
002189	hsa-miR-944	hsa-miR-944,	NA	miR_944_002189	bdnf	-1.5131	-2.5874	-0.4269	0.0065	**0.0286**
002792	hsa-miR-1303	hsa-miR-1303,	NA	miR_1303_002792	bdnf	-1.4148	-2.4301	-0.3889	0.0071	**0.0307**
002199	hsa-miR-125a-3p	hsa-miR-125a-3p,	NA	miR_125a_3p_002199	bdnf	-2.6337	-4.5118	-0.7186	0.0073	**0.0316**
002442	hsa-miR-25#	hsa-miR-25-5p	hsa-miR-25*(17)	miR_25__002442	bdnf	-1.3905	-2.3953	-0.3754	0.0075	**0.0319**
002807	hsa-miR-1270	hsa-miR-1270	NA	miR_1270_002807	bdnf	-1.3664	-2.3560	-0.3668	0.0076	**0.0321**
002360	hsa-miR-491-3p	hsa-miR-491-3p,	mmu-miR-491*(17)	miR_491_3p_002360	bdnf	-1.3622	-2.3509	-0.3635	0.0077	**0.0324**
001129	hsa-miR-137	hsa-miR-137,		miR_137_001129	bdnf	-1.3837	-2.3932	-0.3638	0.0081	**0.0335**
000471	hsa-miR-148b	hsa-miR-148b-3p,	hsa-miR-148b(17),	miR_148b_000471	bdnf	4.6936	1.1680	8.3420	0.0088	**0.0363**
000543	hsa-miR-328	hsa-miR-328-3p,	hsa-miR-328(19),	miR_328_000543	bdnf	3.8335	0.9510	6.7983	0.0089	**0.0366**
002185	hsa-miR-335#	hsa-miR-335-3p,	hsa-miR-335*(17)	miR_335__002185	bdnf	3.9905	0.9808	7.0898	0.0091	**0.0371**
001274	hsa-miR-410	hsa-miR-410-3p,	hsa-miR-410(19),	miR_410_001274	bdnf	4.4149	1.0741	7.8662	0.0093	**0.0376**
002302	hsa-miR-425#	hsa-miR-425-3p,	hsa-miR-425*(17)	miR_425__002302	bdnf	4.2647	1.0232	7.6101	0.0097	**0.0386**
001621	hsa-miR-580	hsa-miR-580-3p,	hsa-miR-580(19)	miR_580_001621	bdnf	-1.3786	-2.4114	-0.3348	0.0099	**0.0391**
000395	hsa-miR-19a	hsa-miR-19a-3p,	hsa-miR-19a(17),	miR_19a_000395	bdnf	3.3893	0.8029	6.0421	0.0100	**0.0395**
002272	hsa-miR-192#	hsa-miR-192-3p,	hsa-miR-192*(17),	miR_192__002272	bdnf	-2.5334	-4.4247	-0.6046	0.0104	**0.0406**
001823	hsa-miR-512-3p	hsa-miR-512-3p,	NA	miR_512_3p_001823	bdnf	-3.8736	-6.7412	-0.9177	0.0107	**0.0414**
001611	hsa-miR-654	hsa-miR-654-5p,	hsa-miR-654(9.2)	miR_654_001611	bdnf	-2.6866	-4.7033	-0.6273	0.0109	**0.0420**
000405	hsa-miR-26a	hsa-miR-26a-5p,	hsa-miR-26a(17),	miR_26a_000405	bdnf	3.3581	0.7560	6.0275	0.0112	**0.0429**
000436	hsa-miR-99b	hsa-miR-99b-5p,	hsa-miR-99b(17),	miR_99b_000436	bdnf	3.2467	0.7189	5.8379	0.0116	**0.0441**
002216	hsa-miR-128a	hsa-miR-128-3p,	hsa-miR-128(19),hsa-miR-128a(10),hsa-miR-128b(10),	miR_128a_002216	bdnf	4.2126	0.9187	7.6140	0.0119	**0.0448**
000390	hsa-miR-15b	hsa-miR-15b-5p,	hsa-miR-15b(17),	miR_15b_000390	bdnf	4.2326	0.9170	7.6571	0.0121	**0.0451**
002358	hsa-miR-489	hsa-miR-489-3p,	hsa-miR-489(19),	miR_489_002358	bdnf	-1.7609	-3.1216	-0.3811	0.0126	**0.0466**
002432	hsa-miR-625#	hsa-miR-625-3p	hsa-miR-625*(17)	miR_625__002432	bdnf	5.7642	1.2157	10.5170	0.0126	**0.0466**
002422	hsa-miR-18a	hsa-miR-18a-5p,	hsa-miR-18a(17),	miR_18a_002422	bdnf	3.5981	0.7542	6.5224	0.0129	**0.0472**
000528	hsa-miR-301	hsa-miR-301a-3p,	hsa-miR-301(9.2),hsa-miR-301a(17),	miR_301_000528	bdnf	3.7086	0.7453	6.7591	0.0139	0.0505

002681	hsa-miR-665	hsa-miR-665,	NA	miR_665_002681	bdnf	-1.2689	-2.2690	-0.2585	0.0141	0.0507
002868	hsa-miR-1249	hsa-miR-1249-3p,	hsa-miR-1249(20),	miR_1249_002868	bdnf	-1.3683	-2.4555	-0.2691	0.0149	0.0534
000545	hsa-miR-331	hsa-miR-331-3p,	hsa-miR-331(9.2),	miR_331_000545	bdnf	2.9560	0.5486	5.4209	0.0159	0.0558
001624	hsa-miR-584	hsa-miR-584-5p,	hsa-miR-584(17),	miR_584_001624	bdnf	-1.4299	-2.5748	-0.2715	0.0158	0.0558
002326	hsa-miR-216b	hsa-miR-216b-5p,	hsa-miR-216b(19),	miR_216b_002326	bdnf	-1.5486	-2.7890	-0.2925	0.0159	0.0558
002270	hsa-miR-183#	hsa-miR-183-3p,	hsa-miR-183*(17),	miR_183__002270	bdnf	-3.1485	-5.6448	-0.5861	0.0164	0.0572
000411	hsa-miR-28	hsa-miR-28-5p,	hsa-miR-28(9.2),	miR_28_000411	bdnf	4.0138	0.7184	7.4171	0.0167	0.0577
001187	hsa-miR-140	hsa-miR-140-5p,		miR_140_001187	bdnf	3.5358	0.6287	6.5270	0.0169	0.0579
002125	hsa-miR-374a#	hsa-miR-374a-3p,	hsa-miR-374a*(17)	miR_374a__002125	bdnf	-1.2580	-2.2780	-0.2273	0.0170	0.0579
002157	hsa-miR-337-3p	hsa-miR-337-3p,	NA	miR_337_3p_002157	bdnf	2.2443	0.3957	4.1271	0.0172	0.0583
	hsa-miR-133a	hsa-miR-133a-3p,	hsa-miR-133a(19),	miR_133a_002246	bdnf	4.6885	0.8083	8.7181	0.0175	0.0589
000502	hsa-miR-200a	hsa-miR-200a-3p,	hsa-miR-200a(17),	miR_200a_000502	bdnf	-1.5329	-2.7806	-0.2692	0.0177	0.0591
001608	hsa-miR-449b	hsa-miR-449b-5p,	hsa-miR-449b(17),	miR_449b_001608	bdnf	-1.4064	-2.5589	-0.2403	0.0183	0.0608
				miR_643_001594	bdnf	-1.2564	-2.2907	-0.2111	0.0187	0.0617
001090	hsa-miR-93	hsa-miR-93-5p,	hsa-miR-93(17),	miR_93_001090	bdnf	2.7964	0.4507	5.1968	0.0193	0.0632
001273	hsa-miR-362	hsa-miR-362-5p,	hsa-miR-362(9.2)	miR_362_001273	bdnf	2.9264	0.4506	5.4632	0.0203	0.0662
000186	hsa-miR-96	hsa-miR-96-5p,	hsa-miR-96(17),	miR_96_000186	bdnf	-3.1272	-5.7040	-0.4800	0.0210	0.0679
000554	hsa-miR-361	hsa-miR-361-5p,	hsa-miR-361(9.2),	miR_361_000554	bdnf	4.1104	0.6026	7.7405	0.0213	0.0685
002116	hsa-miR-361-3p	hsa-miR-361-3p,	NA	miR_361_3p_002116	bdnf	-1.3159	-2.4226	-0.1966	0.0214	0.0685
000533	hsa-miR-302c	hsa-miR-302c-3p,	hsa-miR-302c(17)	miR_302c_000533	bdnf	-1.3118	-2.4205	-0.1905	0.0221	0.0698
002186	hsa-miR-345	hsa-miR-345-5p,	hsa-miR-345(17)	miR_345_002186	bdnf	3.4658	0.4920	6.5276	0.0221	0.0698
002257	hsa-miR-339-5p	hsa-miR-339-5p,	NA	miR_339_5p_002257	bdnf	7.6972	1.0310	14.8034	0.0230	0.0722
000443	hsa-miR-107	hsa-miR-107,		miR_107_000443	bdnf	4.2999	0.5660	8.1724	0.0237	0.0734
002338	hsa-miR-483-5p	hsa-miR-483-5p	NA	miR_483_5p_002338	bdnf	-5.8046	-10.5569	-0.7999	0.0237	0.0734
				miR_1267_002885	bdnf	-1.2224	-2.2713	-0.1622	0.0240	0.0740
002222	hsa-miR-1	hsa-miR-1-3p,	hsa-miR-1(20),	miR_1_002222	bdnf	2.8215	0.3569	5.3467	0.0247	0.0755
002282	hsa-let-7g	hsa-let-7g-5p,	hsa-let-7g(17),	let_7g_002282	bdnf	3.2217	0.4021	6.1205	0.0249	0.0758
002844	hsa-miR-320B	hsa-miR-320b,	NA	miR_320B_002844	bdnf	3.1482	0.3887	5.9836	0.0252	0.0761
002087	hsa-miR-505#	hsa-miR-505-5p	hsa-miR-505*(17)	miR_505__002087	bdnf	3.4297	0.4073	6.5431	0.0259	0.0779
001630	hsa-miR-491	hsa-miR-491-5p,	mmu-miR-491(17)	miR_491_001630	bdnf	4.1315	0.4792	7.9165	0.0263	0.0783
002420	hsa-miR-16-1#	hsa-miR-16-1-3p,	hsa-miR-16-1*(17),	miR_16_1__002420	bdnf	2.5658	0.2998	4.8830	0.0263	0.0783
000382	hsa-let-7f	hsa-let-7f-5p,	hsa-let-7f(17),	let_7f_000382	bdnf	7.4740	0.7887	14.6028	0.0279	0.0819
000399	hsa-miR-23a	hsa-miR-23a-3p,	hsa-miR-23a(17),	miR_23a_000399	bdnf	5.5478	0.5928	10.7470	0.0278	0.0819
002249	hsa-miR-143	hsa-miR-143-3p,	hsa-miR-143(17),	miR_143_002249	bdnf	3.4177	0.3533	6.5758	0.0286	0.0836
002410	hsa-miR-550	hsa-miR-550a-5p,	hsa-miR-550(15),hsa-miR-550a(17),	miR_550_002410	bdnf	-2.8455	-5.3272	-0.2987	0.0289	0.0838
002240	hsa-miR-542-5p	hsa-miR-542-5p,	NA	miR_542_5p_002240	bdnf	-1.7669	-3.3316	-0.1769	0.0296	0.0855
002421	hsa-miR-17#	hsa-miR-17-3p,	hsa-miR-17*(17),	miR_17__002421	bdnf	-1.2081	-2.2902	-0.1140	0.0306	0.0874
002677	hsa-miR-590-3P	hsa-miR-590-3p,	NA	miR_590_3P_002677	bdnf	3.6008	0.3336	6.9744	0.0306	0.0874

000518	hsa-miR-215	hsa-miR-215-5p,	hsa-miR-215(19),	miR_215_000518	bdnf	-4.7945	-8.9634	-0.4347	0.0316	0.0896
002271	hsa-miR-185	hsa-miR-185-5p,	hsa-miR-185(17),	miR_185_002271	bdnf	2.7653	0.2159	5.3796	0.0334	0.0942
000522	hsa-miR-219	hsa-miR-219a-5p,	hsa-miR-219(9.2),hsa-miR-219-5p(19),	miR_219_000522	bdnf	-1.2238	-2.3463	-0.0884	0.0348	0.0974
002901	hsa-miR-1302	hsa-miR-1302,	NA	miR_1302_002901	bdnf	-1.2247	-2.3487	-0.0878	0.0349	0.0974
001614	hsa-miR-572	hsa-miR-572,	NA	miR_572_001614	bdnf	-4.6674	-8.8239	-0.3215	0.0356	0.0990
002289	hsa-miR-139-5p		hsa-miR-139-5p(18)	miR_139_5p_002289	bdnf	2.8986	0.1804	5.6906	0.0365	0.1008
000563	hsa-miR-374	hsa-miR-374a-5p,	hsa-miR-374(9.2),hsa-miR-374a(17),	miR_374_000563	bdnf	2.6066	0.1531	5.1201	0.0372	0.1022
000573	hsa-miR-383	hsa-miR-383-5p,	hsa-miR-383(19),	miR_383_000573	bdnf	-1.1851	-2.2882	-0.0695	0.0374	0.1023
000379	hsa-let-7c	hsa-let-7c-5p,	hsa-let-7c(19),	let_7c_000379	bdnf	3.1869	0.1711	6.2935	0.0382	0.1038
001120	hsa-miR-520f	hsa-miR-520f-3p,	hsa-miR-520f(19),	miR_520f_001120	bdnf	-1.8391	-3.5559	-0.0917	0.0393	0.1055
002441	hsa-miR-24-2#	hsa-miR-24-2-5p	hsa-miR-24-2*(17)	miR_24_2__002441	bdnf	2.6547	0.1308	5.2423	0.0392	0.1055
002791	hsa-miR-1244	hsa-miR-1244,	NA	miR_1244_002791	bdnf	-1.3003	-2.5257	-0.0594	0.0401	0.1072
001566	hsa-miR-603	hsa-miR-603,	NA	miR_603_001566	bdnf	-2.5394	-4.9086	-0.1111	0.0405	0.1078
000498	hsa-miR-199a	hsa-miR-199a-5p,	hsa-miR-199a(9.2),	miR_199a_000498	bdnf	3.6344	0.1298	7.2616	0.0420	0.1111
002231	hsa-miR-9#	hsa-miR-9-3p,	hsa-miR-9*(17),	miR_9__002231	bdnf	3.0845	0.0562	6.2045	0.0459	0.1201
002429	hsa-miR-548c-5p	hsa-miR-548am-5p,hsa-miR-548c-5p,hsa-miR-548o-5p,	NA	miR_548c_5p_002429	bdnf	1.8279	0.0352	3.6528	0.0456	0.1201
002838	hsa-miR-1291	hsa-miR-1291,	NA	miR_1291_002838	bdnf	-2.8379	-5.5665	-0.0306	0.0476	0.1240
002248	hsa-miR-142-5p	hsa-miR-142-5p,		miR_142_5p_002248	bdnf	3.1978	0.0098	6.4875	0.0493	0.1278
002021	hsa-miR-674		hsa-miR-674(10),	miR_674_002021	bdnf	-2.0434	-4.0437	-0.0014	0.0498	0.1285
000452	hsa-miR-127	hsa-miR-127-3p,	hsa-miR-127(9.2),	miR_127_000452	bdnf	3.7120	-0.0264	7.5902	0.0517	0.1320
002193	hsa-miR-886-5p	NA	hsa-miR-886-5p(15),	miR_886_5p_002193	bdnf	3.0485	-0.0223	6.2136	0.0517	0.1320
002278	hsa-miR-145	hsa-miR-145-5p,	hsa-miR-145(17),	miR_145_002278	bdnf	4.4927	-0.0728	9.2669	0.0538	0.1363
002789	hsa-miR-1269	hsa-miR-1269a	hsa-miR-1269(17)	miR_1269_002789	bdnf	-1.0301	-2.0668	0.0176	0.0539	0.1363
002089	hsa-miR-505	hsa-miR-505-3p,	hsa-miR-505(17),	miR_505_002089	bdnf	2.8623	-0.0627	5.8730	0.0552	0.1388
002269	hsa-miR-183	hsa-miR-183-5p,	hsa-miR-183(17),	miR_183_002269	bdnf	-1.0750	-2.1733	0.0357	0.0578	0.1445
001052	hsa-miR-508	hsa-miR-508-3p	hsa-miR-508(9.2)	miR_508_001052	bdnf	-1.8674	-3.7747	0.0776	0.0597	0.1488
002308	hsa-miR-17	hsa-miR-17-5p,	hsa-miR-17(17),	miR_17_002308	bdnf	2.4600	-0.1279	5.1148	0.0626	0.1550
002285	hsa-miR-186	hsa-miR-186-5p,	hsa-miR-186(17),	miR_186_002285	bdnf	2.8208	-0.1501	5.8801	0.0629	0.1551
002238	hsa-miR-411#	hsa-miR-411-3p,	hsa-miR-411*(17),	miR_411__002238	bdnf	-0.9858	-2.0203	0.0596	0.0644	0.1563
002244	hsa-miR-455-3p	hsa-miR-455-3p,	ssc-miR-455(18)	miR_455_3p_002244	bdnf	-1.1660	-2.3881	0.0715	0.0646	0.1563
002266	hsa-miR-545#	hsa-miR-545-5p	hsa-miR-545*(17)	miR_545__002266	bdnf	-1.3130	-2.6826	0.0759	0.0638	0.1563
002346	hsa-miR-551b#	hsa-miR-551b-5p	hsa-miR-551b*(17)	miR_551b__002346	bdnf	-2.3875	-4.8526	0.1414	0.0640	0.1563
002398	hsa-miR-579	hsa-miR-579-3p,	hsa-miR-579(19)	miR_579_002398	bdnf	2.3769	-0.1582	4.9764	0.0663	0.1595
000451	hsa-miR-126#	hsa-miR-126-5p,	hsa-miR-126*(17),	miR_126__000451	bdnf	3.5567	-0.2494	7.5081	0.0673	0.1608
001174	hsa-miR-525	hsa-miR-525-5p,	hsa-miR-525(9.2)	miR_525_001174	bdnf	-1.4091	-2.8975	0.1022	0.0674	0.1608

000431	hsa-miR-92a	hsa-miR-92a-3p,	hsa-miR-92(5),hsa-miR-92a(17),	miR_92a_000431	bdnf	2.0464	-0.1527	4.2940	0.0683	0.1621
001514	hsa-miR-659	hsa-miR-659-3p	hsa-miR-659(17)	miR_659_001514	bdnf	-1.9645	-4.0461	0.1622	0.0699	0.1652
000604	hsa-miR-424	hsa-miR-424-5p,	hsa-miR-424(17),	miR_424_000604	bdnf	2.3324	-0.1996	4.9286	0.0712	0.1674
000389	hsa-miR-15a	hsa-miR-15a-5p,	hsa-miR-15a(17),	miR_15a_000389	bdnf	4.3266	-0.3820	9.2577	0.0722	0.1682
002182	hsa-miR-939	hsa-miR-939-5p,	hsa-miR-939(18)	miR_939_002182	bdnf	-1.3172	-2.7336	0.1200	0.0722	0.1682
001156	hsa-miR-518b	hsa-miR-518b,	NA	miR_518b_001156	bdnf	-2.2770	-4.7267	0.2356	0.0753	0.1746
000587	hsa-miR-29c	hsa-miR-29c-3p,	hsa-miR-29c(17),	miR_29c_000587	bdnf	2.4857	-0.2736	5.3214	0.0778	0.1795
002331	hsa-miR-409-5p	hsa-miR-409-5p,		miR_409_5p_002331	bdnf	1.6437	-0.1889	3.5101	0.0789	0.1814
002112	hsa-miR-29a	hsa-miR-29a-3p,	hsa-miR-29a(17),	miR_29a_002112	bdnf	3.1852	-0.3766	6.8744	0.0801	0.1833
001821	hsa-miR-484	hsa-miR-484,	NA	miR_484_001821	bdnf	4.2725	-0.5195	9.2953	0.0812	0.1846
002323	hsa-miR-454	hsa-miR-454-3p,	hsa-miR-454(17),	miR_454_002323	bdnf	3.2259	-0.3968	6.9804	0.0814	0.1846
002279	hsa-miR-31	hsa-miR-31-5p	hsa-miR-31(17)	miR_31_002279	bdnf	-3.5106	-7.3228	0.4585	0.0822	0.1847
002333	hsa-miR-181c#	hsa-miR-181c-3p	hsa-miR-181c*(17)	miR_181c__002333	bdnf	-1.6768	-3.5343	0.2164	0.0821	0.1847
000422	hsa-miR-30e-3p	hsa-miR-30e-3p,	hsa-miR-30e*(17),	miR_30e_3p_000422	bdnf	3.5706	-0.4668	7.7719	0.0836	0.1871
001097	hsa-miR-146b	hsa-miR-146b-5p,	hsa-miR-146b(9.2),	miR_146b_001097	bdnf	2.2620	-0.3009	4.8907	0.0840	0.1871
002384	hsa-miR-519b-3p	hsa-miR-519b-3p,	NA	miR_519b_3p_002384	bdnf	-1.2456	-2.6452	0.1741	0.0851	0.1888
000397	hsa-miR-21	hsa-miR-21-5p,	hsa-miR-21(17),	miR_21_000397	bdnf	3.9876	-0.5784	8.7634	0.0877	0.1937
002404	hsa-let-7b#	hsa-let-7b-3p,	hsa-let-7b*(17),	let_7b__002404	bdnf	-1.6174	-3.4547	0.2548	0.0898	0.1976
001043	hsa-miR-497	hsa-miR-497-5p,	hsa-miR-497(17),	miR_497_001043	bdnf	-1.0036	-2.1569	0.1634	0.0915	0.2002
001277	hsa-miR-485-3p	hsa-miR-485-3p,	NA	miR_485_3p_001277	bdnf	3.3241	-0.5387	7.3370	0.0924	0.2014
001613	hsa-miR-571	hsa-miR-571,	NA	miR_571_001613	bdnf	-1.6197	-3.4773	0.2737	0.0930	0.2019
				miR_767_3p_001995	bdnf	-1.1392	-2.4724	0.2122	0.0979	0.2117
000482	hsa-miR-181c	hsa-miR-181c-5p,	hsa-miR-181c(17),	miR_181c_000482	bdnf	2.6603	-0.4913	5.9117	0.0987	0.2124
002419	hsa-miR-15a#	hsa-miR-15a-3p,	hsa-miR-15a*(17),	miR_15a__002419	bdnf	1.8719	-0.3549	4.1485	0.0998	0.2140
000417	hsa-miR-30a-5p	hsa-miR-30a-5p,	hsa-miR-30a(17),	miR_30a_5p_000417	bdnf	3.4130	-0.6588	7.6518	0.1013	0.2144
001027	hsa-miR-432#	hsa-miR-432-3p	hsa-miR-432*(17)	miR_432__001027	bdnf	-1.6254	-3.5365	0.3235	0.1014	0.2144
001534	hsa-miR-567	hsa-miR-567,	NA	miR_567_001534	bdnf	-1.6252	-3.5343	0.3217	0.1011	0.2144
002170	hsa-miR-106a#	hsa-miR-106a-3p	hsa-miR-106a*(17)	miR_106a__002170	bdnf	-1.6256	-3.5388	0.3254	0.1017	0.2144
001141	hsa-miR-451	hsa-miR-451a,	hsa-miR-451(17),	miR_451_001141	bdnf	-3.3107	-7.1481	0.6853	0.1030	0.2145
002109	hsa-miR-32	hsa-miR-32-5p,	hsa-miR-32(17),	miR_32_002109	bdnf	2.5679	-0.5109	5.7420	0.1028	0.2145
002313	hsa-miR-139-3p		hsa-miR-139-3p(18)	miR_139_3p_002313	bdnf	4.8950	-0.9593	11.0953	0.1027	0.2145
000534	hsa-miR-302c#	hsa-miR-302c-5p,	hsa-miR-302c*(17)	miR_302c__000534	bdnf	-3.8280	-8.2632	0.8216	0.1050	0.2176
002136	hsa-miR-33a#	hsa-miR-33a-3p,	hsa-miR-33a*(17),	miR_33a__002136	bdnf	1.3829	-0.2986	3.0929	0.1073	0.2216
002166	hsa-miR-29b-2#	hsa-miR-29b-2-5p,	hsa-miR-29b-2*(17),	miR_29b_2__002166	bdnf	-1.6104	-3.5428	0.3606	0.1084	0.2216
002208	hsa-miR-450b-3p	hsa-miR-450b-3p,	NA	miR_450b_3p_002208	bdnf	-1.6302	-3.5864	0.3657	0.1085	0.2216
002433	hsa-miR-628-5p	hsa-miR-628-5p,	NA	miR_628_5p_002433	bdnf	-7.6905	-16.3022	1.8073	0.1090	0.2216
002874	hsa-miR-1304	hsa-miR-1304-5p	hsa-miR-1304(17)	miR_1304_002874	bdnf	-1.6309	-3.5933	0.3714	0.1095	0.2216

				miR_548E_002881	bdnf	-1.1244	-2.4841	0.2543	0.1092	0.2216
002148	hsa-miR-144#	hsa-miR-144-5p	hsa-miR-144*(17)	miR_144__002148	bdnf	-3.1080	-6.8011	0.7314	0.1111	0.2239
002769	hsa-miR-1227	hsa-miR-1227-3p	hsa-miR-1227(18)	miR_1227_002769	bdnf	1.4730	-0.3406	3.3196	0.1118	0.2244
000469	hsa-miR-147	hsa-miR-147a,	hsa-miR-147(17),	miR_147_000469	bdnf	-1.5497	-3.4366	0.3741	0.1135	0.2269
002414	hsa-miR-616	hsa-miR-616-3p,	hsa-miR-616(17)	miR_616_002414	bdnf	-0.8933	-1.9907	0.2164	0.1140	0.2271
001014	hsa-miR-20b	hsa-miR-20b-5p,	hsa-miR-20b(17),	miR_20b_001014	bdnf	2.5348	-0.6293	5.7996	0.1173	0.2326
002217	hsa-miR-18b	hsa-miR-18b-5p,	hsa-miR-18b(17)	miR_18b_002217	bdnf	-1.9279	-4.2922	0.4947	0.1177	0.2326
002880	hsa-miR-1208	hsa-miR-1208,	NA	miR_1208_002880	bdnf	-1.3231	-2.9595	0.3410	0.1183	0.2329
000268	hsa-miR-7	hsa-miR-7-5p,	hsa-miR-7(17),	miR_7_000268	bdnf	1.7828	-0.4683	4.0848	0.1212	0.2377
002149	hsa-miR-145#	hsa-miR-145-3p,	hsa-miR-145*(17)	miR_145__002149	bdnf	-1.4325	-3.2225	0.3905	0.1225	0.2394
000602	hsa-miR-30b	hsa-miR-30b-5p,	hsa-miR-30b(17),	miR_30b_000602	bdnf	3.6229	-0.9793	8.4390	0.1244	0.2421
000377	hsa-let-7a	hsa-let-7a-5p,	hsa-let-7a(17),	let_7a_000377	bdnf	5.8159	-1.6055	13.7971	0.1273	0.2459
002317	hsa-miR-181a-2#	hsa-miR-181a-2-3p	hsa-miR-181a-2*(17)	miR_181a_2__002317	bdnf	2.3890	-0.6731	5.5455	0.1272	0.2459
002237	hsa-miR-548d-5p	hsa-miR-548d-5p	NA	miR_548d_5p_002237	bdnf	1.2756	-0.3751	2.9537	0.1303	0.2507
001992	hsa-miR-668	hsa-miR-668-3p,	hsa-miR-668(19)	miR_668_001992	bdnf	-1.2950	-2.9517	0.3899	0.1309	0.2509
002250	hsa-miR-193a-3p	hsa-miR-193a-3p,		miR_193a_3p_002250	bdnf	-1.3758	-3.1482	0.4291	0.1340	0.2559
000480	hsa-miR-181a	hsa-miR-181a-5p,	hsa-miR-181a(17),	miR_181a_000480	bdnf	3.3184	-1.0200	7.8470	0.1355	0.2576
000497	hsa-miR-197	hsa-miR-197-3p,	hsa-miR-197(17),	miR_197_000497	bdnf	6.4257	-1.9514	15.5187	0.1362	0.2576
001159	hsa-miR-518d	hsa-miR-518d-3p,	hsa-miR-518d(9.2)	miR_518d_001159	bdnf	-1.5544	-3.5649	0.4980	0.1364	0.2576
000433	hsa-miR-95	hsa-miR-95-3p,	hsa-miR-95(19),	miR_95_000433	bdnf	-2.5186	-5.7608	0.8350	0.1390	0.2616
001280	hsa-miR-455	hsa-miR-455-5p,	hsa-miR-455(9.2),	miR_455_001280	bdnf	-1.1183	-2.5886	0.3741	0.1409	0.2623
001606	hsa-miR-661	hsa-miR-661	NA	miR_661_001606	bdnf	-0.9812	-2.2737	0.3284	0.1410	0.2623
002161	hsa-miR-324-3p	hsa-miR-324-3p,	NA	miR_324_3p_002161	bdnf	2.3572	-0.7667	5.5795	0.1403	0.2623
001278	hsa-miR-486	hsa-miR-486-5p,	hsa-miR-486(9.2),	miR_486_001278	bdnf	-2.0759	-4.7845	0.7099	0.1424	0.2630
002296	hsa-miR-885-5p	hsa-miR-885-5p,	NA	miR_885_5p_002296	bdnf	3.1186	-1.0284	7.4393	0.1422	0.2630
000407	hsa-miR-26b	hsa-miR-26b-5p,	hsa-miR-26b(17),	miR_26b_000407	bdnf	1.6423	-0.5733	3.9072	0.1471	0.2707
001543	hsa-miR-589	hsa-miR-589-3p	hsa-miR-589(9.2),hsa-miR-589*(17)	miR_589_001543	bdnf	-1.0644	-2.4909	0.3830	0.1483	0.2711
002218	hsa-miR-10b	hsa-miR-10b-5p,	hsa-miR-10b(17),	miR_10b_002218	bdnf	-3.1489	-7.2602	1.1446	0.1479	0.2711
000409	hsa-miR-27b	hsa-miR-27b-3p,	hsa-miR-27b(17),	miR_27b_000409	bdnf	2.2431	-0.8396	5.4216	0.1552	0.2825
002352	hsa-miR-652	hsa-miR-652-3p,	hsa-miR-652(17),	miR_652_002352	bdnf	2.8537	-1.1215	6.9888	0.1614	0.2928
001279	hsa-miR-487a	hsa-miR-487a-3p,	hsa-miR-487(7.1),hsa-miR-487a(19)	miR_487a_001279	bdnf	-1.1353	-2.7233	0.4785	0.1665	0.3010
002234	hsa-miR-140-3p	hsa-miR-140-3p,		miR_140_3p_002234	bdnf	2.2452	-0.9454	5.5386	0.1694	0.3052
002390	hsa-miR-219-2-3p	hsa-miR-219a-2-3p,	hsa-miR-219-2-3p(19),	miR_219_2_3p_002390	bdnf	-1.2370	-2.9892	0.5468	0.1725	0.3096
000570	hsa-miR-380-5p	hsa-miR-380-5p	hsa-miR-380*(17),	miR_380_5p_000570	bdnf	-1.2363	-2.9920	0.5511	0.1736	0.3105
000419	hsa-miR-30c	hsa-miR-30c-5p,	hsa-miR-30c(17),	miR_30c_000419	bdnf	4.0383	-1.7471	10.1643	0.1746	0.3106

000442	hsa-miR-106b	hsa-miR-106b-5p,	hsa-miR-106b(17),	miR_106b_000442	bdnf	1.4945	-0.6597	3.6954	0.1749	0.3106
002829	hsa-miR-1200	hsa-miR-1200,	NA	miR_1200_002829	bdnf	-1.2350	-2.9975	0.5596	0.1757	0.3111
002114	hsa-miR-130b#	hsa-miR-130b-5p	hsa-miR-130b*(17)	miR_130b__002114	bdnf	1.1642	-0.5245	2.8815	0.1773	0.3121
002196	hsa-miR-99b#	hsa-miR-99b-3p,	hsa-miR-99b*(17),	miR_99b__002196	bdnf	2.4005	-1.0738	5.9969	0.1776	0.3121
002094	hsa-miR-218-1#	hsa-miR-218-1-3p	hsa-miR-218-1*(17)	miR_218_1__002094	bdnf	-1.2332	-3.0047	0.5707	0.1786	0.3128
002247	hsa-miR-133b	hsa-miR-133b,		miR_133b_002247	bdnf	-1.0502	-2.5665	0.4898	0.1798	0.3139
000500	hsa-miR-199b	hsa-miR-199b-5p,	hsa-miR-199b(9.2),	miR_199b_000500	bdnf	2.1240	-0.9734	5.3182	0.1806	0.3142
002436	hsa-miR-629	hsa-miR-629-5p	hsa-miR-629(17)	miR_629_002436	bdnf	1.6207	-0.7494	4.0475	0.1814	0.3144
000457	hsa-miR-132	hsa-miR-132-3p,	hsa-miR-132(17),	miR_132_000457	bdnf	3.0718	-1.5249	7.8831	0.1932	0.3304
000561	hsa-miR-373	hsa-miR-373-3p,	hsa-miR-373(17)	miR_373_000561	bdnf	-1.2998	-3.2267	0.6654	0.1929	0.3304
002245	hsa-miR-122	hsa-miR-122-5p,	hsa-miR-122(17),	miR_122_002245	bdnf	2.1830	-1.0832	5.5571	0.1921	0.3304
002336	hsa-miR-196a#	hsa-miR-196a-3p	hsa-miR-196a*(17)	miR_196a__002336	bdnf	-1.2250	-3.0386	0.6226	0.1919	0.3304
002233	hsa-miR-331-5p	hsa-miR-331-5p,	NA	miR_331_5p_002233	bdnf	-1.2967	-3.2283	0.6733	0.1950	0.3325
002895	hsa-miR-720	NA	hsa-miR-720(18),	miR_720_002895	bdnf	1.5241	-0.7885	3.8906	0.1977	0.3359
002341	hsa-miR-708	hsa-miR-708-5p,	hsa-miR-708(17),	miR_708_002341	bdnf	1.9208	-1.0150	4.9437	0.2015	0.3412
001111	hsa-miR-511	hsa-miR-511-5p,	hsa-miR-511(19)	miR_511_001111	bdnf	1.5551	-0.8370	4.0048	0.2040	0.3431
001338	rno-miR-7#	hsa-miR-7-1-3p,	hsa-miR-7-1*(17),	miR_7__001338	bdnf	2.4852	-1.3273	6.4451	0.2039	0.3431
002138	hsa-miR-92a-2#	hsa-miR-92a-2-5p	hsa-miR-92a-2*(17)	miR_92a_2__002138	bdnf	-1.2131	-3.0882	0.6983	0.2114	0.3527
002172	hsa-let-7i#	hsa-let-7i-3p,	hsa-let-7i*(17),	let_7i__002172	bdnf	1.4888	-0.8407	3.8731	0.2117	0.3527
002840	hsa-miR-1275	hsa-miR-1275,	NA	miR_1275_002840	bdnf	-1.2129	-3.0890	0.6996	0.2117	0.3527
002354	hsa-miR-871	mmu-miR-871-5p	hsa-miR-871(10),	miR_871_002354	bdnf	-1.2106	-3.0985	0.7140	0.2155	0.3577
001562	hsa-miR-629	hsa-miR-629-3p	hsa-miR-629(9.2),hsa-miR-629*(17)	miR_629_001562	bdnf	1.7288	-1.0019	4.5349	0.2164	0.3581
000400	hsa-miR-23b	hsa-miR-23b-3p,	hsa-miR-23b(17),	miR_23b_000400	bdnf	-1.5868	-4.0812	0.9726	0.2215	0.3653
002329	hsa-miR-452	hsa-miR-452-5p,	hsa-miR-452(17),	miR_452_002329	bdnf	1.0460	-0.6415	2.7620	0.2253	0.3705
001173	hsa-miR-524	hsa-miR-524-3p,	hsa-miR-524(9.2)	miR_524_001173	bdnf	-1.2035	-3.1288	0.7601	0.2273	0.3709
002387	hsa-miR-518f#	hsa-miR-518f-5p	hsa-miR-518f*(17)	miR_518f__002387	bdnf	-1.2040	-3.1266	0.7567	0.2265	0.3709
002851	hsa-miR-1278	hsa-miR-1278	NA	miR_1278_002851	bdnf	-1.2032	-3.1300	0.7619	0.2278	0.3709
001582	hsa-miR-638	hsa-miR-638	NA	miR_638_001582	bdnf	2.9153	-1.8068	7.8646	0.2299	0.3731
000470	hsa-miR-148a	hsa-miR-148a-3p,	hsa-miR-148(3),hsa-miR-148a(17),	miR_148a_000470	bdnf	3.7526	-2.3258	10.2092	0.2311	0.3739
002092	hsa-miR-508-5p	hsa-miR-508-5p	NA	miR_508_5p_002092	bdnf	-1.2002	-3.1427	0.7811	0.2327	0.3741
002339	hsa-miR-483-3p	hsa-miR-483-3p	NA	miR_483_3p_002339	bdnf	-0.9445	-2.4772	0.6123	0.2324	0.3741
001558	hsa-miR-601	hsa-miR-601,	NA	miR_601_001558	bdnf	-3.1870	-8.2395	2.1436	0.2357	0.3765
002263	hsa-miR-190b	hsa-miR-190b,	mmu-miR-190b(17)	miR_190b_002263	bdnf	-1.5256	-3.9995	1.0120	0.2357	0.3765
001109	hsa-miR-502	hsa-miR-502-5p,	hsa-miR-502(9.2)	miR_502_001109	bdnf	1.6592	-1.0879	4.4826	0.2385	0.3786
001625	hsa-miR-585	hsa-miR-585-3p	hsa-miR-585(19)	miR_585_001625	bdnf	-1.5424	-4.0571	1.0382	0.2384	0.3786
002884	hsa-miR-1274B	NA	hsa-miR-1274b(16),	miR_1274B_002884	bdnf	1.5177	-1.0133	4.1136	0.2417	0.3825

000580	hsa-miR-20a	hsa-miR-20a-5p,	hsa-miR-20(6),hsa-miR-20a(17),	miR_20a_000580	bdnf	1.4882	-1.0128	4.0523	0.2453	0.3870
002107	hsa-miR-195#	hsa-miR-195-3p,	hsa-miR-195*(17),	miR_195__002107	bdnf	-1.1904	-3.1849	0.8452	0.2491	0.3918
000477	hsa-miR-154	hsa-miR-154-5p,	hsa-miR-154(17),	miR_154_000477	bdnf	-0.7399	-1.9927	0.5289	0.2512	0.3938
002276	hsa-miR-222	hsa-miR-222-3p,	hsa-miR-222(17),	miR_222_002276	bdnf	2.0622	-1.4529	5.7027	0.2531	0.3955
000516	hsa-miR-213	hsa-miR-181a-3p,	hsa-miR-181a*(17),hsa-miR-213(8),	miR_213_000516	bdnf	1.3062	-0.9326	3.5955	0.2544	0.3965
002215	hsa-miR-196b	hsa-miR-196b-5p,	hsa-miR-196b(17),	miR_196b_002215	bdnf	1.6789	-1.2000	4.6417	0.2553	0.3966
000403	hsa-miR-25	hsa-miR-25-3p,	hsa-miR-25(17),	miR_25_000403	bdnf	1.3204	-0.9765	3.6706	0.2616	0.4051
001284	hsa-miR-542-3p	hsa-miR-542-3p,	NA	miR_542_3p_001284	bdnf	1.3546	-1.0132	3.7791	0.2640	0.4076
002355	hsa-miR-532-3p	hsa-miR-532-3p,	NA	miR_532_3p_002355	bdnf	1.6350	-1.2372	4.5908	0.2669	0.4108
002132	hsa-miR-132#	hsa-miR-132-5p,	hsa-miR-132*(17),	miR_132__002132	bdnf	-1.2633	-3.4620	0.9855	0.2679	0.4111
002239	hsa-miR-654-3p	hsa-miR-654-3p,	NA	miR_654_3p_002239	bdnf	0.9440	-0.7315	2.6478	0.2706	0.4140
000460	hsa-miR-135a	hsa-miR-135a-5p,	hsa-miR-135(3),hsa-miR-135a(17),	miR_135a_000460	bdnf	1.7543	-1.3850	4.9935	0.2761	0.4212
000420	hsa-miR-30d	hsa-miR-30d-5p,	hsa-miR-30d(17),	miR_30d_000420	bdnf	1.5233	-1.2136	4.3359	0.2776	0.4222
002443	hsa-miR-26a-1#	hsa-miR-26a-1-3p,	hsa-miR-26a-1*(17),	miR_26a_1__002443	bdnf	1.3191	-1.1034	3.8009	0.2879	0.4352
002843	hsa-miR-1276	hsa-miR-1276,	NA	miR_1276_002843	bdnf	-1.3262	-3.7255	1.1329	0.2871	0.4352
002267	hsa-miR-545	hsa-miR-545-3p,	hsa-miR-545(17)	miR_545_002267	bdnf	1.2081	-1.0393	3.5066	0.2939	0.4431
001319	hsa-miR-374-5p	hsa-miR-374b-5p,	hsa-miR-374b(17),	miR_374_5p_001319	bdnf	2.3994	-2.0986	7.1040	0.3002	0.4506
002386	hsa-miR-523	hsa-miR-523-3p,	hsa-miR-523(17)	miR_523_002386	bdnf	-2.4049	-6.8077	2.2059	0.3007	0.4506
002428	hsa-miR-500	hsa-miR-500a-5p,	hsa-miR-500(15),hsa-miR-500a(17),	miR_500_002428	bdnf	1.1499	-1.0318	3.3797	0.3034	0.4533
002417	hsa-let-7f-1#	hsa-let-7f-1-3p,	hsa-let-7f-1*(17),	let_7f_1__002417	bdnf	-0.8616	-2.5200	0.8249	0.3140	0.4678
002297	hsa-miR-422a	hsa-miR-422a,	NA	miR_422a_002297	bdnf	1.2486	-1.1858	3.7431	0.3170	0.4708
002425	hsa-miR-19b-1#	hsa-miR-19b-1-5p,	hsa-miR-19b-1*(17),	miR_19b_1__002425	bdnf	-0.6665	-1.9750	0.6594	0.3222	0.4758
				miR_1204_002872	bdnf	-0.9135	-2.6995	0.9052	0.3220	0.4758
002083	hsa-miR-502-3p	hsa-miR-502-3p,	NA	miR_502_3p_002083	bdnf	1.2108	-1.1854	3.6651	0.3242	0.4773
000515	hsa-miR-212	hsa-miR-212-3p,	hsa-miR-212(17),	miR_212_000515	bdnf	2.0392	-1.9962	6.2408	0.3261	0.4785
002402	hsa-miR-517a	hsa-miR-517a-3p,hsa-miR-517b-3p,	hsa-miR-517a(17)	miR_517a_002402	bdnf	-1.4463	-4.2794	1.4706	0.3269	0.4785
001612	hsa-miR-655	hsa-miR-655-3p,	hsa-miR-655(19)	miR_655_001612	bdnf	1.1072	-1.1190	3.3835	0.3317	0.4814
002097	hsa-miR-222#	hsa-miR-222-5p	hsa-miR-222*(17)	miR_222__002097	bdnf	-0.8199	-2.4535	0.8409	0.3305	0.4814
002777	hsa-miR-1178	hsa-miR-1178-3p,	hsa-miR-1178(18)	miR_1178_002777	bdnf	-1.0216	-3.0541	1.0536	0.3314	0.4814
001605	hsa-miR-548d	hsa-miR-548d-3p	hsa-miR-548d(9.2)	miR_548d_001605	bdnf	-0.9337	-2.8132	0.9821	0.3364	0.4855
002251	hsa-miR-200b	hsa-miR-200b-3p,	hsa-miR-200b(17),	miR_200b_002251	bdnf	0.9993	-1.0287	3.0689	0.3359	0.4855
002275	hsa-miR-370	hsa-miR-370-3p,	hsa-miR-370(19),	miR_370_002275	bdnf	2.6608	-2.7205	8.3398	0.3384	0.4870
000391	hsa-miR-16	hsa-miR-16-5p,	hsa-miR-16(17),	miR_16_000391	bdnf	1.2123	-1.2852	3.7731	0.3438	0.4934
001990	hsa-miR-758	hsa-miR-758-3p,	hsa-miR-758(18),	miR_758_001990	bdnf	2.3147	-2.4519	7.3142	0.3464	0.4958

000565	hsa-miR-376a	hsa-miR-376a-3p,	hsa-miR-376a(17),	miR_376a_000565	bdnf	2.9073	-3.1056	9.2934	0.3501	0.4972
002361	hsa-miR-146b-3p	hsa-miR-146b-3p,	NA	miR_146b_3p_002361	bdnf	1.0236	-1.1162	3.2098	0.3504	0.4972
002824	hsa-miR-1292	hsa-miR-1292-5p,	hsa-miR-1292(18)	miR_1292_002824	bdnf	-0.8290	-2.5441	0.9163	0.3490	0.4972
002763	hsa-miR-1228#	hsa-miR-1228-5p	hsa-miR-1228*(17)	miR_1228__002763	bdnf	-0.8229	-2.5487	0.9334	0.3555	0.5030
001271	hsa-miR-363	hsa-miR-363-3p,	hsa-miR-363(17),	miR_363_001271	bdnf	-2.1648	-6.6305	2.5145	0.3578	0.5049
000572	hsa-miR-382	hsa-miR-382-5p,	hsa-miR-382(17),	miR_382_000572	bdnf	3.2760	-3.6313	10.6784	0.3607	0.5068
002115	hsa-miR-26a-2#	hsa-miR-26a-2-3p,	hsa-miR-26a-2*(17)	miR_26a_2__002115	bdnf	1.3170	-1.4927	4.2068	0.3611	0.5068
002243	hsa-miR-378		hsa-miR-378(17),hsa-miR-378a-3p(19),	miR_378_002243	bdnf	-1.4170	-4.4134	1.6734	0.3641	0.5096
000577	hsa-miR-98	hsa-miR-98-5p,	hsa-miR-98(18),	miR_98_000577	bdnf	2.8302	-3.2476	9.2898	0.3685	0.5143
000510	hsa-miR-206	hsa-miR-206,		miR_206_000510	bdnf	-1.1561	-3.6441	1.3962	0.3706	0.5150
002658	hsa-miR-338-5P			miR_338_5P_002658	bdnf	-1.2617	-3.9750	1.5283	0.3711	0.5150
000464	hsa-miR-142-3p	hsa-miR-142-3p,		miR_142_3p_000464	bdnf	1.2794	-1.5360	4.1752	0.3761	0.5205
000571	hsa-miR-381	hsa-miR-381-3p,	hsa-miR-381(18),	miR_381_000571	bdnf	2.4858	-2.9792	8.2587	0.3791	0.5230
001604	hsa-miR-651	hsa-miR-651-5p	hsa-miR-651(19)	miR_651_001604	bdnf	-0.7138	-2.2914	0.8892	0.3799	0.5230
002380	hsa-miR-106b#	hsa-miR-106b-3p,	hsa-miR-106b*(17),	miR_106b__002380	bdnf	1.5196	-1.8533	5.0083	0.3809	0.5230
001178	hsa-let-7d#	hsa-let-7d-3p,	hsa-let-7d*(17),	let_7d__001178	bdnf	-0.8080	-2.5984	1.0154	0.3820	0.5230
000529	hsa-miR-302a	hsa-miR-302a-3p,	hsa-miR-302(3.1),hsa-miR-302a(17),	miR_302a_000529	bdnf	-1.4804	-4.7430	1.8941	0.3847	0.5253
000521	hsa-miR-218	hsa-miR-218-5p,	hsa-miR-218(17),	miR_218_000521	bdnf	-1.1882	-3.8516	1.5489	0.3905	0.5292
002439	hsa-miR-23a#	hsa-miR-23a-5p,	hsa-miR-23a*(17),	miR_23a__002439	bdnf	0.7202	-0.9228	2.3905	0.3917	0.5292
002783	hsa-miR-548J	hsa-miR-548j-5p	hsa-miR-548j(19)	miR_548J_002783	bdnf	0.7315	-0.9289	2.4196	0.3893	0.5292
002896	hsa-miR-1260	hsa-miR-1260a,	hsa-miR-1260(17),	miR_1260_002896	bdnf	2.0052	-2.5311	6.7526	0.3916	0.5292
002776	hsa-miR-1179	hsa-miR-1179,	NA	miR_1179_002776	bdnf	0.7170	-0.9318	2.3932	0.3955	0.5330
002779	hsa-miR-1271	hsa-miR-1271-5p,	hsa-miR-1271(17),	miR_1271_002779	bdnf	1.0281	-1.3568	3.4708	0.4006	0.5384
002252	hsa-miR-338-3p	hsa-miR-338-3p,	tgu-miR-338(15)	miR_338_3p_002252	bdnf	0.7135	-0.9584	2.4137	0.4044	0.5421
002400	hsa-miR-520c-3p	hsa-miR-520c-3p,	NA	miR_520c_3p_002400	bdnf	3.0137	-4.0004	10.5404	0.4085	0.5461
002254	hsa-miR-151-3p	hsa-miR-151a-3p,	hsa-miR-151-3p(17),	miR_151_3p_002254	bdnf	2.4730	-3.3827	8.6835	0.4151	0.5534
002268	hsa-miR-874	hsa-miR-874-3p,	hsa-miR-874(19),	miR_874_002268	bdnf	-0.7635	-2.5977	1.1053	0.4200	0.5585
002860	hsa-miR-1252	hsa-miR-1252-5p	hsa-miR-1252(19)	miR_1252_002860	bdnf	-0.9840	-3.3509	1.4409	0.4224	0.5602
001589	hsa-miR-616	hsa-miR-616-5p	hsa-miR-616(9.2),hsa-miR-616*(17)	miR_616_001589	bdnf	0.6684	-0.9694	2.3333	0.4253	0.5612
001597	hsa-miR-645	hsa-miR-645,	NA	miR_645_001597	bdnf	-1.2894	-4.3941	1.9160	0.4253	0.5612
001535	hsa-miR-551b	hsa-miR-551b-3p,	hsa-miR-551b(17),	miR_551b_001535	bdnf	1.6541	-2.4886	5.9727	0.4389	0.5766
002678	hsa-miR-191#	hsa-miR-191-3p,	hsa-miR-191*(17)	miR_191__002678	bdnf	0.7243	-1.1040	2.5865	0.4393	0.5766
002212	hsa-miR-888	hsa-miR-888-5p,	hsa-miR-888(17)	miR_888_002212	bdnf	1.8239	-2.7743	6.6394	0.4426	0.5794
001116	hsa-miR-520b	hsa-miR-520b,		miR_520b_001116	bdnf	1.5140	-2.3334	5.5130	0.4452	0.5813
001996	hsa-miR-454#	hsa-miR-454-5p	hsa-miR-454*(17)	miR_454__001996	bdnf	0.8605	-1.3668	3.1382	0.4513	0.5878

002130	hsa-miR-122#	hsa-miR-122-3p,	hsa-miR-122*(17)	miR_122__002130	bdnf	0.6326	-1.0199	2.3126	0.4546	0.5907
002847	hsa-miR-1180	hsa-miR-1180-3p,	hsa-miR-1180(19)	miR_1180_002847	bdnf	0.8332	-1.3757	3.0916	0.4621	0.5988
001818	rno-miR-29c#	hsa-miR-29c-5p,	hsa-miR-29c*(17),	miR_29c__001818	bdnf	0.8366	-1.3899	3.1134	0.4638	0.5996
001020	hsa-miR-365	hsa-miR-365a-3p,hsa-miR-365b-3p,	hsa-miR-365(17),	miR_365_001020	bdnf	1.1312	-1.9190	4.2763	0.4709	0.6071
002117	hsa-miR-362-3p	hsa-miR-362-3p,	NA	miR_362_3p_002117	bdnf	0.9149	-1.5630	3.4551	0.4720	0.6071
001028	hsa-miR-433	hsa-miR-433-3p,	hsa-miR-433(19),	miR_433_001028	bdnf	1.0422	-1.8551	4.0250	0.4841	0.6211
002388	hsa-miR-518f	hsa-miR-518f-3p,	hsa-miR-518f(17)	miR_518f_002388	bdnf	-1.2505	-4.7072	2.3316	0.4881	0.6246
001551	hsa-miR-597	hsa-miR-597-5p,	hsa-miR-597(19)	miR_597_001551	bdnf	-1.0605	-4.1396	2.1176	0.5079	0.6467
002623	hsa-miR-155	hsa-miR-155-5p,	hsa-miR-155(17),	miR_155_002623	bdnf	1.6571	-3.1723	6.7274	0.5073	0.6467
002798	hsa-miR-548P	hsa-miR-548p,	NA	miR_548P_002798	bdnf	-0.9645	-3.7982	1.9527	0.5122	0.6505
000387	hsa-miR-10a	hsa-miR-10a-5p,	hsa-miR-10a(17),	miR_10a_000387	bdnf	0.9213	-1.8260	3.7455	0.5142	0.6513
000473	hsa-miR-150	hsa-miR-150-5p,	hsa-miR-150(17),	miR_150_000473	bdnf	-1.4504	-5.8331	3.1361	0.5283	0.6676
001024	hsa-miR-429	hsa-miR-429,	mml-miR-429(19)	miR_429_001024	bdnf	0.5032	-1.1310	2.1643	0.5478	0.6792
001510	hsa-miR-656	hsa-miR-656-3p,	hsa-miR-656(19),	miR_656_001510	bdnf	-0.4849	-2.0540	1.1095	0.5482	0.6792
001528	hsa-miR-561	hsa-miR-561-3p	hsa-miR-561(17)	miR_561_001528	bdnf	-0.5755	-2.4121	1.2957	0.5433	0.6792
001599	hsa-miR-646	hsa-miR-646,	NA	miR_646_001599	bdnf	-1.0842	-4.5533	2.5111	0.5488	0.6792
002090	hsa-miR-513-5p	hsa-miR-513a-5p,	hsa-miR-513-5p(10)	miR_513_5p_002090	bdnf	-0.5757	-2.4120	1.2951	0.5431	0.6792
002096	hsa-miR-221#	hsa-miR-221-5p,	hsa-miR-221*(17)	miR_221__002096	bdnf	0.5103	-1.1220	2.1695	0.5416	0.6792
002347	hsa-miR-570	hsa-miR-570-3p	hsa-miR-570(17)	miR_570_002347	bdnf	0.5103	-1.1220	2.1695	0.5416	0.6792
002818	hsa-miR-1254	hsa-miR-1254,	NA	miR_1254_002818	bdnf	0.4983	-1.1327	2.1561	0.5509	0.6792
002842	hsa-miR-1184	hsa-miR-1184,	NA	miR_1184_002842	bdnf	-0.5691	-2.4155	1.3122	0.5500	0.6792
002857	hsa-miR-663B	hsa-miR-663b,	NA	miR_663B_002857	bdnf	-0.5926	-2.4916	1.3433	0.5451	0.6792
001979	hsa-miR-431	hsa-miR-431-5p,	hsa-miR-431(17),	miR_431_001979	bdnf	-0.6139	-2.6211	1.4346	0.5534	0.6807
002801	hsa-miR-1255B	hsa-miR-1255b-5p,	hsa-miR-1255b(17)	miR_1255B_002801	bdnf	0.6653	-1.5534	2.9341	0.5591	0.6860
001515	hsa-miR-660	hsa-miR-660-5p,	hsa-miR-660(17),	miR_660_001515	bdnf	0.6826	-1.6032	3.0216	0.5608	0.6865
002211	hsa-miR-220c	NA	hsa-miR-220c(15),	miR_220c_002211	bdnf	-0.5572	-2.4217	1.3429	0.5622	0.6865
000491	hsa-miR-192	hsa-miR-192-5p,	hsa-miR-192(17),	miR_192_000491	bdnf	1.1338	-2.6805	5.0976	0.5648	0.6880
000494	hsa-miR-195	hsa-miR-195-5p,	hsa-miR-195(17),	miR_195_000494	bdnf	0.8270	-1.9921	3.7272	0.5685	0.6909
000489	hsa-miR-190	hsa-miR-190a-5p,	hsa-miR-190(17),hsa-miR-190a(19),	miR_190_000489	bdnf	0.7916	-1.9865	3.6484	0.5797	0.6912
000574	hsa-miR-384	hsa-miR-384,	NA	miR_384_000574	bdnf	-0.5419	-2.4298	1.3825	0.5777	0.6912
001619	hsa-miR-578	hsa-miR-578,	NA	miR_578_001619	bdnf	-0.5411	-2.4302	1.3845	0.5785	0.6912
002205	hsa-miR-876-5p	hsa-miR-876-5p,	NA	miR_876_5p_002205	bdnf	-0.5411	-2.4302	1.3846	0.5786	0.6912
002310	hsa-miR-18b#	hsa-miR-18b-3p	hsa-miR-18b*(17)	miR_18b__002310	bdnf	-0.5453	-2.4280	1.3736	0.5742	0.6912
002330	hsa-miR-452#	hsa-miR-452-3p	hsa-miR-452*(17)	miR_452__002330	bdnf	-0.5422	-2.4296	1.3818	0.5775	0.6912
002405	hsa-let-7c#	NA	hsa-let-7c*(16)	let_7c__002405	bdnf	-0.5442	-2.4286	1.3766	0.5754	0.6912
002870	hsa-miR-1248	hsa-miR-1248,	NA	miR_1248_002870	bdnf	-0.5439	-2.4287	1.3775	0.5757	0.6912
002108	hsa-miR-30c-1#	hsa-miR-30c-1-3p,	hsa-miR-30c-1*(17),	miR_30c_1__002108	bdnf	-0.5358	-2.4331	1.3984	0.5839	0.6947

001048	hsa-miR-503	hsa-miR-503-5p,	hsa-miR-503(18),	miR_503_001048	bdnf	0.6840	-1.7607	3.1895	0.5861	0.6957
000583	hsa-miR-9	hsa-miR-9-5p,	hsa-miR-9(17),	miR_9_000583	bdnf	1.0700	-2.8482	5.1463	0.5971	0.7038
002002	hsa-miR-770-5p	hsa-miR-770-5p,	NA	miR_770_5p_002002	bdnf	-0.6986	-3.2381	1.9076	0.5952	0.7038
002137	hsa-miR-92a-1#	hsa-miR-92a-1-5p,	hsa-miR-92a-1*(17)	miR_92a_1__002137	bdnf	0.6936	-1.8501	3.3032	0.5958	0.7038
001590	hsa-miR-548c	hsa-miR-548c-3p,	hsa-miR-548c(9.2)	miR_548c_001590	bdnf	-0.7531	-3.5168	2.0898	0.5992	0.7046
002357	hsa-miR-488	hsa-miR-488-3p,	hsa-miR-488(17),	miR_488_002357	bdnf	-0.5166	-2.4434	1.4484	0.6031	0.7069
002423	hsa-miR-18a#	hsa-miR-18a-3p	hsa-miR-18a*(17)	miR_18a__002423	bdnf	-0.6163	-2.9146	1.7364	0.6039	0.7069
000413	hsa-miR-29b	hsa-miR-29b-3p,	hsa-miR-29b(17),	miR_29b_000413	bdnf	0.7869	-2.2708	3.9402	0.6174	0.7071
001113	hsa-miR-517#	hsa-miR-517-5p,	hsa-miR-517*(17)	miR_517__001113	bdnf	-0.5016	-2.4516	1.4873	0.6178	0.7071
001184	hsa-miR-129-3p	hsa-miR-129-2-3p,	hsa-miR-129-3p(17),	miR_129_3p_001184	bdnf	-0.4984	-2.4534	1.4959	0.6211	0.7071
001516	hsa-miR-425-5p	hsa-miR-425-5p,	hsa-miR-425(17),	miR_425_5p_001516	bdnf	1.5343	-4.4021	7.8394	0.6197	0.7071
001518	hsa-miR-532	hsa-miR-532-5p,	hsa-miR-532(9.2),	miR_532_001518	bdnf	0.8753	-2.5059	4.3737	0.6157	0.7071
001539	hsa-miR-586	hsa-miR-586,	NA	miR_586_001539	bdnf	-0.6646	-3.2464	1.9862	0.6191	0.7071
001546	hsa-miR-592	hsa-miR-592,	mml-miR-592(19)	miR_592_001546	bdnf	-0.5006	-2.4522	1.4899	0.6188	0.7071
001559	hsa-miR-626	hsa-miR-626,	NA	miR_626_001559	bdnf	-0.5017	-2.4516	1.4871	0.6178	0.7071
001586	hsa-miR-613	hsa-miR-613,	NA	miR_613_001586	bdnf	-0.5016	-2.4516	1.4873	0.6178	0.7071
001591	hsa-miR-617	hsa-miR-617,	NA	miR_617_001591	bdnf	-0.4985	-2.4534	1.4956	0.6210	0.7071
001982	hsa-miR-524-5p	hsa-miR-524-5p,	hsa-miR-524*(9.2)	miR_524_5p_001982	bdnf	-0.5016	-2.4517	1.4875	0.6179	0.7071
002183	hsa-miR-941	hsa-miR-941	NA	miR_941_002183	bdnf	-0.4972	-2.4541	1.4990	0.6222	0.7071
002904	hsa-miR-548L	hsa-miR-548l,	NA	miR_548L_002904	bdnf	-0.6601	-3.2037	1.9503	0.6161	0.7071
002427	hsa-miR-499-3p	hsa-miR-499a-3p,	hsa-miR-499-3p(17),	miR_499_3p_002427	bdnf	-0.4883	-2.4590	1.5222	0.6309	0.7154
002309	hsa-miR-424#	hsa-miR-424-3p,	hsa-miR-424*(17),	miR_424__002309	bdnf	-0.4507	-2.2818	1.4146	0.6328	0.7159
000555	hsa-miR-367	hsa-miR-367-3p,	hsa-miR-367(17),	miR_367_000555	bdnf	-1.3832	-7.0170	4.5920	0.6420	0.7247
001555	hsa-miR-623	hsa-miR-623	NA	miR_623_001555	bdnf	1.1566	-3.6797	6.2358	0.6449	0.7264
001538	hsa-miR-548a	hsa-miR-548a-3p,	hsa-miR-548a(9.2)	miR_548a_001538	bdnf	-0.6787	-3.5971	2.3280	0.6539	0.7349
001046	hsa-miR-500	hsa-miR-500a-3p	hsa-miR-500(9.2),hsa-miR-500*(15),hsa-miR-500a*(17)	miR_500_001046	bdnf	-0.4343	-2.3874	1.5579	0.6662	0.7471
000508	hsa-miR-204	hsa-miR-204-5p,	hsa-miR-204(17),	miR_204_000508	bdnf	0.7861	-2.8429	4.5507	0.6750	0.7553
002334	hsa-miR-182	hsa-miR-182-5p,	hsa-miR-182(17)	miR_182_002334	bdnf	-0.5874	-3.3218	2.2244	0.6783	0.7573
002173	hsa-miR-15b#	hsa-miR-15b-3p,	hsa-miR-15b*(17),	miR_15b__002173	bdnf	-0.6642	-3.7655	2.5372	0.6800	0.7575
000449	hsa-miR-125b	hsa-miR-125b-5p,	hsa-miR-125b(17),	miR_125b_000449	bdnf	0.5544	-2.0821	3.2619	0.6828	0.7576
000567	hsa-miR-378	hsa-miR-378a-5p,	hsa-miR-378(9.2),hsa-miR-378*(17),	miR_378_000567	bdnf	-0.8424	-4.7958	3.2751	0.6831	0.7576
000426	hsa-miR-34a	hsa-miR-34a-5p,	hsa-miR-34a(17),	miR_34a_000426	bdnf	-1.6109	-9.1445	6.5475	0.6889	0.7625
000493	hsa-miR-194	hsa-miR-194-5p,	hsa-miR-194(17),	miR_194_000493	bdnf	0.8007	-3.1222	4.8826	0.6932	0.7655
001593	hsa-miR-618	hsa-miR-618,	NA	miR_618_001593	bdnf	-1.2230	-7.3775	5.3404	0.7072	0.7793
002300	hsa-miR-200c	hsa-miR-200c-3p,	hsa-miR-200c(17),	miR_200c_002300	bdnf	0.6201	-2.6309	3.9796	0.7117	0.7825
000512	hsa-miR-210	hsa-miR-210-3p,	hsa-miR-210(19),	miR_210_000512	bdnf	1.2608	-5.4630	8.4629	0.7203	0.7903

002447	hsa-miR-29a#	hsa-miR-29a-5p,	hsa-miR-29a*(17),	miR_29a__002447	bdnf	-0.4883	-3.2504	2.3526	0.7327	0.8022
002194	hsa-miR-886-3p	NA	hsa-miR-886-3p(15),	miR_886_3p_002194	bdnf	0.5473	-2.5806	3.7757	0.7345	0.8024
000560	hsa-miR-372	hsa-miR-372-3p,	hsa-miR-372(19),	miR_372_000560	bdnf	-0.3386	-2.4593	1.8282	0.7568	0.8233
002408	hsa-miR-548b-5p	hsa-miR-548b-5p	NA	miR_548b_5p_002408	bdnf	-0.5062	-3.6579	2.7486	0.7569	0.8233
002113	hsa-miR-31#	hsa-miR-31-3p	hsa-miR-31*(17)	miR_31__002113	bdnf	-0.3571	-2.6993	2.0413	0.7677	0.8333
002284	hsa-miR-138	hsa-miR-138-5p,	hsa-miR-138(17),	miR_138_002284	bdnf	-0.4502	-3.5717	2.7725	0.7809	0.8459
001102	hsa-miR-376b	hsa-miR-376b-3p,	hsa-miR-376b(18),	miR_376b_001102	bdnf	0.3830	-2.3505	3.1930	0.7857	0.8493
000463	hsa-miR-141	hsa-miR-141-3p,	hsa-miR-141(17),	miR_141_000463	bdnf	-0.3276	-2.7531	2.1585	0.7937	0.8560
002883	hsa-miR-1274A	NA	hsa-miR-1274a(16)	miR_1274A_002883	bdnf	-0.6186	-5.2789	4.2710	0.7997	0.8607
000437	hsa-miR-100	hsa-miR-100-5p,	hsa-miR-100(17),	miR_100_000437	bdnf	0.5741	-3.9365	5.2965	0.8065	0.8661
002445	hsa-miR-27a#	hsa-miR-27a-5p,	hsa-miR-27a*(17),	miR_27a__002445	bdnf	0.3364	-2.3589	3.1060	0.8087	0.8667
002260	hsa-miR-342-3p	hsa-miR-342-3p,	NA	miR_342_3p_002260	bdnf	0.8185	-5.9059	8.0234	0.8166	0.8734
001560	hsa-miR-627	hsa-miR-627-5p,	hsa-miR-627(19)	miR_627_001560	bdnf	-0.3072	-3.0233	2.4849	0.8268	0.8820
002228	hsa-miR-126	hsa-miR-126-3p,	hsa-miR-126(17),	miR_126_002228	bdnf	0.3887	-3.0871	3.9891	0.8289	0.8820
002363	hsa-miR-202	hsa-miR-202-3p,	hsa-miR-202(17)	miR_202_002363	bdnf	-0.2362	-2.3856	1.9605	0.8311	0.8820
002367	hsa-miR-193b	hsa-miR-193b-3p,	hsa-miR-193b(17),	miR_193b_002367	bdnf	0.2978	-2.4206	3.0920	0.8317	0.8820
000416	hsa-miR-30a-3p	hsa-miR-30a-3p,	hsa-miR-30a(3.1),hsa-miR-30a*(17),	miR_30a_3p_000416	bdnf	-0.3884	-4.2120	3.5879	0.8452	0.8835
000564	hsa-miR-375	hsa-miR-375,		miR_375_000564	bdnf	-0.3521	-3.8084	3.2284	0.8444	0.8835
001153	hsa-miR-517c	hsa-miR-517c-3p,	hsa-miR-517c(17),	miR_517c_001153	bdnf	-0.2564	-2.7184	2.2679	0.8401	0.8835
001578	hsa-miR-635	hsa-miR-635,	NA	miR_635_001578	bdnf	-0.2505	-2.7274	2.2896	0.8447	0.8835
001592	hsa-miR-642	hsa-miR-642a-5p,	hsa-miR-642(15),hsa-miR-642a(17)	miR_642_001592	bdnf	-0.3447	-3.5859	3.0055	0.8375	0.8835
002316	hsa-miR-34a#	hsa-miR-34a-3p	hsa-miR-34a*(17)	miR_34a__002316	bdnf	-0.3311	-3.5876	3.0354	0.8446	0.8835
002397	hsa-miR-518a-3p	hsa-miR-518a-3p,	NA	miR_518a_3p_002397	bdnf	-0.2567	-2.7972	2.3502	0.8449	0.8835
002187	hsa-miR-942	hsa-miR-942-5p,	hsa-miR-942(19)	miR_942_002187	bdnf	0.4460	-4.2432	5.3649	0.8550	0.8918
002235	hsa-miR-509-5p	hsa-miR-509-5p,	eca-miR-509-5p(20)	miR_509_5p_002235	bdnf	-0.1875	-2.2360	1.9039	0.8589	0.8941
001563	hsa-miR-630	hsa-miR-630,	NA	miR_630_001563	bdnf	-0.2223	-2.8017	2.4256	0.8675	0.9012
002369	hsa-miR-515-3p	hsa-miR-515-3p,	NA	miR_515_3p_002369	bdnf	0.2000	-2.3713	2.8390	0.8800	0.9124
000485	hsa-miR-184	hsa-miR-184,		miR_184_000485	bdnf	0.2661	-3.2387	3.8978	0.8834	0.9140
002088	hsa-miR-636	hsa-miR-636	NA	miR_636_002088	bdnf	-0.6310	-8.8704	8.3534	0.8858	0.9146
000398	hsa-miR-22	hsa-miR-22-3p,	hsa-miR-22(17),	miR_22_000398	bdnf	-0.3238	-5.2917	4.9046	0.9008	0.9184
000435	hsa-miR-99a	hsa-miR-99a-5p,	hsa-miR-99a(17),	miR_99a_000435	bdnf	0.2381	-3.2290	3.8294	0.8945	0.9184
000507	hsa-miR-203	hsa-miR-203a-3p,	hsa-miR-203(18),hsa-miR-203a(20),	miR_203_000507	bdnf	0.2184	-3.0803	3.6294	0.8982	0.9184
000514	hsa-miR-211	hsa-miR-211-5p,	hsa-miR-211(17)	miR_211_000514	bdnf	-0.2622	-3.9526	3.5701	0.8912	0.9184
001521	hsa-miR-553	hsa-miR-553,	NA	miR_553_001521	bdnf	0.1579	-2.2683	2.6443	0.8995	0.9184
002306	hsa-miR-214	hsa-miR-214-3p,	hsa-miR-214(17),	miR_214_002306	bdnf	0.2692	-4.0200	4.7500	0.9039	0.9184
002315	hsa-miR-10b#	hsa-miR-10b-3p	hsa-miR-10b*(17)	miR_10b__002315	bdnf	0.2198	-3.0107	3.5579	0.8953	0.9184

002743	hsa-miR-520D-3P	hsa-miR-520d-3p,	NA	miR_520D_3P_002743	bdnf	0.2284	-3.4019	3.9951	0.9034	0.9184
002004	hsa-miR-802	hsa-miR-802,	NA	miR_802_002004	bdnf	-0.1409	-2.4915	2.2665	0.9075	0.9189
002281	hsa-miR-193a-5p	hsa-miR-193a-5p,		miR_193a_5p_002281	bdnf	-0.1784	-3.1622	2.8974	0.9080	0.9189
001602	hsa-miR-649	hsa-miR-649,	NA	miR_649_001602	bdnf	0.1385	-2.2653	2.6015	0.9109	0.9200
000527	hsa-miR-296	hsa-miR-296-5p,	hsa-miR-296(9.2),	miR_296_000527	bdnf	0.2609	-4.4984	5.2574	0.9162	0.9216
002253	hsa-miR-101	hsa-miR-101-3p,	hsa-miR-101(17),	miR_101_002253	bdnf	0.1594	-2.7640	3.1708	0.9159	0.9216
002897	hsa-miR-664	hsa-miR-664a-3p,	hsa-miR-664(17),hsa-miR-664-3p(18),	miR_664_002897	bdnf	0.2095	-4.1588	4.7768	0.9265	0.9302
002129	hsa-miR-30b#	hsa-miR-30b-3p,	hsa-miR-30b*(17)	miR_30b__002129	bdnf	0.1067	-2.2555	2.5260	0.9301	0.9319
001119	hsa-miR-520e	hsa-miR-520e,	NA	miR_520e_001119	bdnf	-0.3080	-7.2069	7.1037	0.9327	0.9327

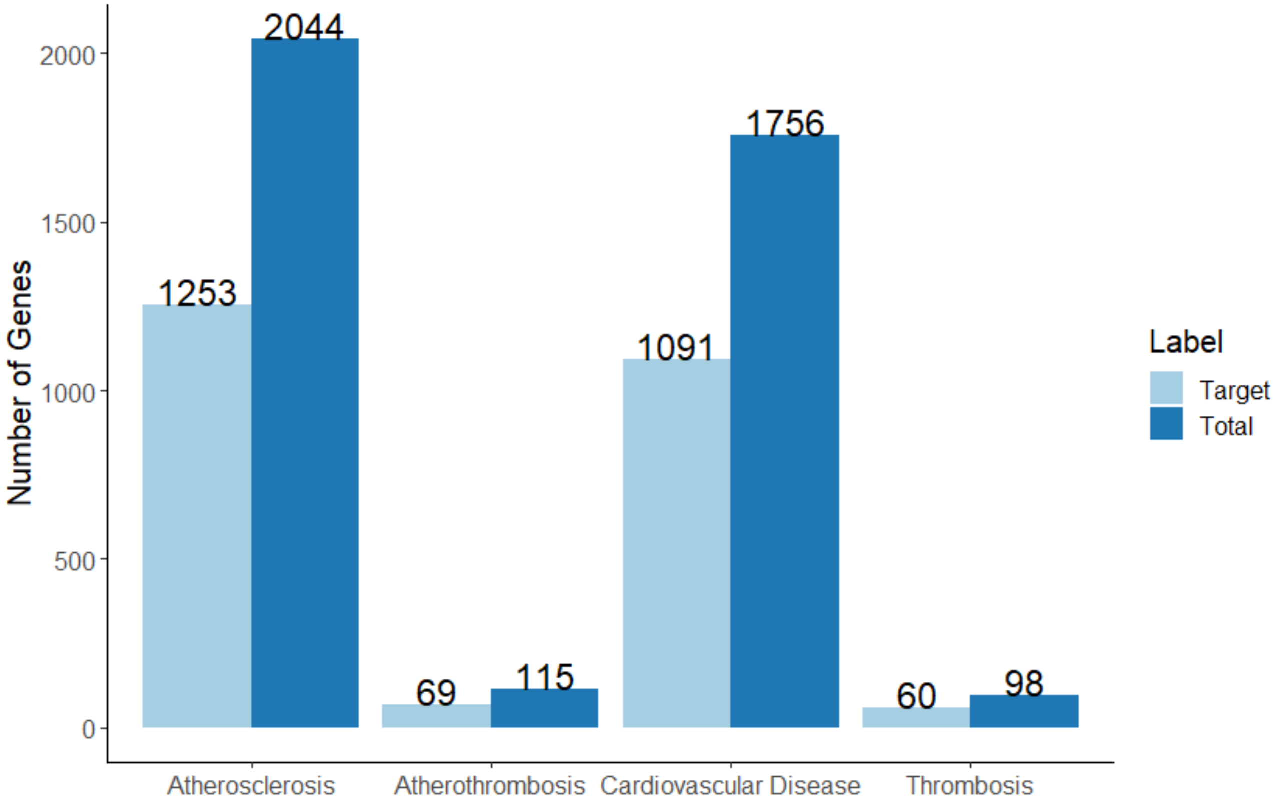

Supplementary Figure 1. The total number of genes associated with atherosclerosis, atherothrombosis, cardiovascular disease, and thrombosis in DisGeNET dataset and the number of genes associated with each disease and targeted by miRNAs associated with BDNF (up-regulated group).

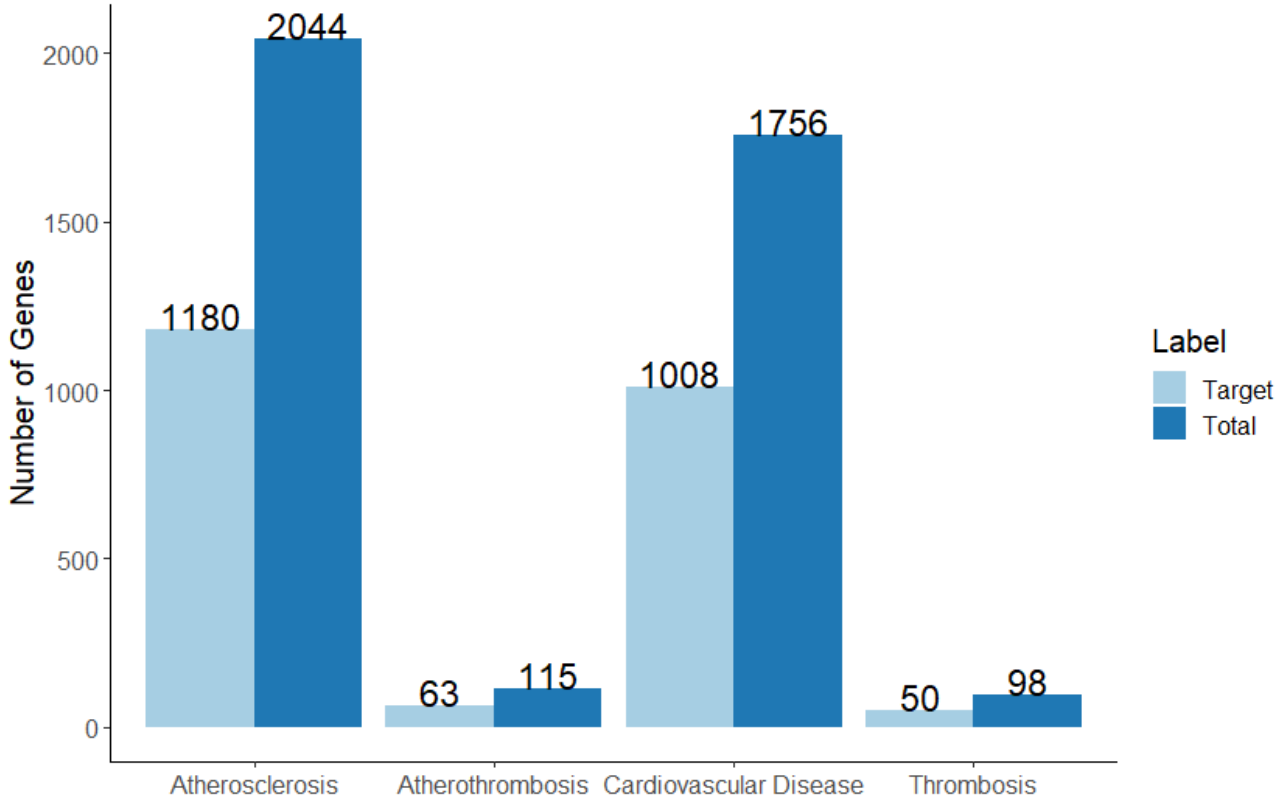

Supplementary Figure 2. The total number of genes associated with atherosclerosis, atherothrombosis, cardiovascular disease, and thrombosis in DisGeNET dataset and the number of genes associated with each disease and targeted by miRNAs associated with BDNF (down-regulated group)

Supplementary Table 4. Genes associated with the group of up- and down-regulated miRNA

Gene	Gene ID	Gene Name	UniProt	Protein Class	Group
**CYP2C19**	1557	Cytochrome P450 family 2 subfamily C member 19	P33261		both
**F3**	2152	Coagulation Cactor III, Tissue Tactor	P13726	Receptor	both
**IL6**	3569	Interleukin 6	P05231		both
**MTHFR**	4524	Methylenetetrahydrofolate Reductase	P42898		both
**PLAT**	5327	Tissue Type Plasminogen Activator	P00750	Enzyme	both
**PTGS2**	5743	Prostaglandin-endoperoxide Synthase 2	P35354	Enzyme	both
**SERPINE1**	5054	serpin family E member 1	P05121	Enzyme modulator	both
**TFPI**	7035	Tissue Factor Pathway Inhibitor	P10646	Enzyme modulator	both
**THBD**	7056	Thrombomodulin	P07204		both
**TNF**	7124	Tumor Necrosis Factor	P01375	Signaling	both
**ACE2**	59272	Angiotensin I Converting Enzyme 2	Q9BYF1	Enzyme	down
**CPB2**	1361	Carboxypeptidase B2	Q96IY4	Enzyme	down
**PTGS1**	5742	Prostaglandin-endoperoxide Synthase 1	P23219	Enzyme	down
**IL1B**	3553	Interleukin 1 beta	P01584		up
**LPA**	4018	Lipoprotein(a)	P08519	Enzyme	up
**PROC**	5624	Protein C, Inactivator of Coagulation Factors Va and VIIIa	P04070	Enzyme	up
**SELP**	6403	P-selectin	P16109		up
**VWF**	7450	Von Willebrand Factor	P04275	Enzyme modulator	up

Both: genes related to both down- and up- regulated miRNAs (dark); down: genes related to down-regulated miRNAs (red); up: genes related to up-regulated miRNAs (blue).

Supplementary Table 5. Genes targeted by up-regulated miRNAs associated with both depression and atherothrombosis.

miRNA	Gene	Depression state	UniProt
hsa-miR-432-5p	ACE	Recurrent depression	P12821
hsa-miR-15b-5p<br>hsa-miR-103a-3p<br>hsa-miR-330-3p<br>hsa-miR-134-5p<br>hsa-miR-495-3p<br>hsa-miR-191-5p<br>hsa-miR-26b-3p	BDNF	Chronic depression<br>Severe depression	P23560
hsa-miR-744-3p	IL18	Severe depression	Q14116
hsa-miR-495-3p<br>hsa-miR-590-5p<br>hsa-miR-30d-3p	IL1B	Recurrent depression<br>Severe depression	P01584
hsa-let-7d-5p<br>hsa-miR-574-3p<br>hsa-let-7e-5p	IL6	clinical depression<br>Mild depression<br>Severe depression	P05231
hsa-miR-15b-5p<br>hsa-miR-24-3p<br>hsa-miR-103a-3p	MTHFR	Severe depression	P42898
hsa-miR-326<br>hsa-miR-340-5p<br>hsa-miR-22-5p	MTR	Severe depression	Q99707
hsa-miR-130a-3p<br>hsa-miR-130b-3p<br>hsa-miR-301b-3p	TNF	Severe depression	P01375

Abbrevations: ACE: Angiotensin I Converting Enzyme; BDNF: Brain Derived Neurotrophic Factor; IL18: Interleukin 18; IL1B: Interleukin 1beta; IL6: Interleukin 6; MTHFR: Methylenetetrahydrofolate Reductase; MTR: Methionine synthase; TNF: Tumor Necrosis Factor.

Supplementary Table 6. Genes targeted by down-regulated miRNAs associated with both depression and atherothrombosis

miRNA	Gene	Depression state	UniProt
hsa-miR-564<br>hsa-miR-604	APOE	Clinical depression<br>Severe depression	P02649
hsa-miR-15b-5p<br>hsa-miR-103a-3p<br>hsa-miR-330-3p<br>hsa-miR-134-5p<br>hsa-miR-495-3p<br>hsa-miR-191-5p<br>hsa-miR-26b-3p	BDNF	Chronic depression<br>Severe depression	P23560
hsa-miR-744-3p	IL18	Severe depression	Q14116
hsa-let-7d-5p<br>hsa-miR-574-3p<br>hsa-let-7e-5p	IL6	clinical depression<br>Mild depression<br>Severe depression	P05231
hsa-miR-15b-5p<br>hsa-miR-24-3p<br>hsa-miR-103a-3p	MTHFR	Severe depression	P42898
hsa-miR-326<br>hsa-miR-340-5p<br>hsa-miR-22-5p	MTR	Severe depression	Q99707
hsa-miR-130a-3p<br>hsa-miR-130b-3p<br>hsa-miR-301b-3p	TNF	Severe depression	P01375

Abbreviations: APOE: Apolipoprotein E; BDNF: Brain Derived Neurotrophic Factor; IL18: Interleukin 18; IL6: Interleukin 6; MTHFR: Methylenetetrahydrofolate Reductase; MTR: Methionine synthase; TNF: Tumor Necrosis Factor.

Supplementary Figure 3. Conceptual diagram of causal mediation analysis, that hypothesized mechanism linking platelets and BDI-II.

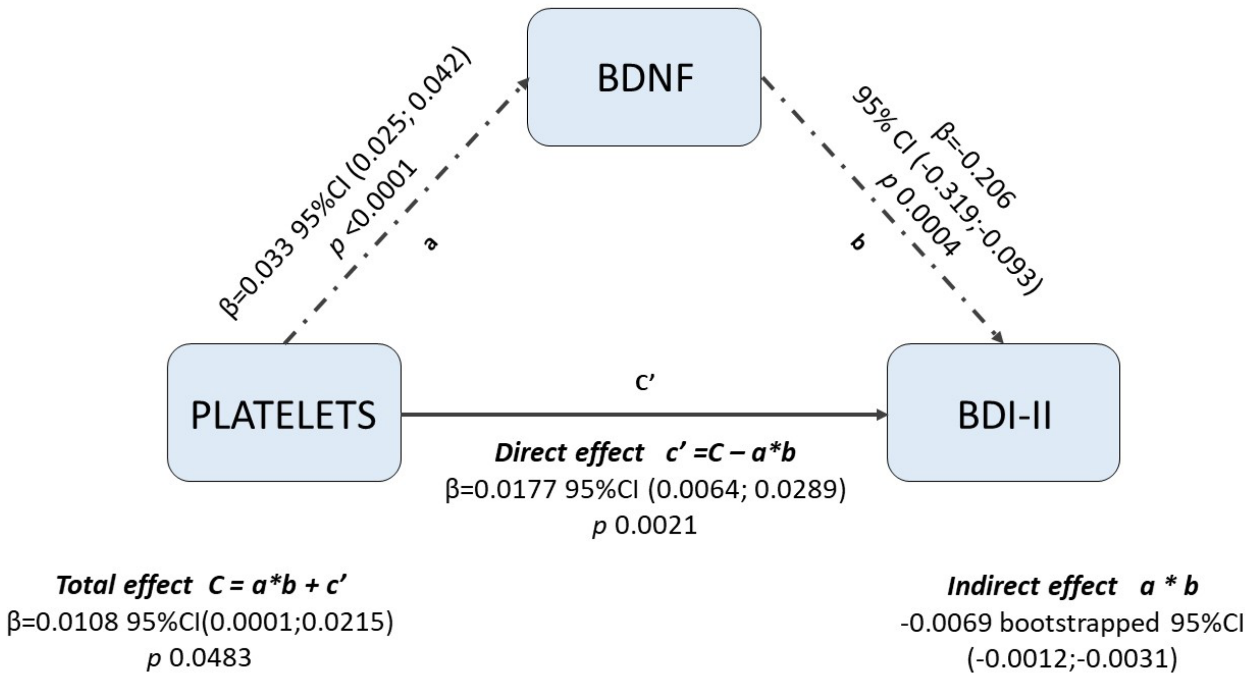

Figure legend. The solid black arrow represents the effect of platelets on BDI-II levels that operates directly or through a pathway different from the mediator analysed in the current study (BDNF). The dotted black arrows represent the suggested alternative pathway, where an indirect effect of platelets on BDI-II is mediated by BDNF levels. BDI: Beck Depression Inventory; BDNF: brain-derived neurotrophic factor.
